# Genome-wide association study of offspring birth weight in 86 577 women identifies five novel loci and highlights maternal genetic effects that are independent of fetal genetics

**DOI:** 10.1093/hmg/ddx429

**Published:** 2018-01-03

**Authors:** Robin N Beaumont, Nicole M Warrington, Alana Cavadino, Jessica Tyrrell, Michael Nodzenski, Momoko Horikoshi, Frank Geller, Ronny Myhre, Rebecca C Richmond, Lavinia Paternoster, Jonathan P Bradfield, Eskil Kreiner-Møller, Ville Huikari, Sarah Metrustry, Kathryn L Lunetta, Jodie N Painter, Jouke-Jan Hottenga, Catherine Allard, Sheila J Barton, Ana Espinosa, Julie A Marsh, Catherine Potter, Ge Zhang, Wei Ang, Diane J Berry, Luigi Bouchard, Shikta Das, Early Growth Genetics (EGG) Consortium, Hakon Hakonarson, Jani Heikkinen, Øyvind Helgeland, Berthold Hocher, Albert Hofman, Hazel M Inskip, Samuel E Jones, Manolis Kogevinas, Penelope A Lind, Letizia Marullo, Sarah E Medland, Anna Murray, Jeffrey C Murray, Pål R Njølstad, Ellen A Nohr, Christoph Reichetzeder, Susan M Ring, Katherine S Ruth, Loreto Santa-Marina, Denise M Scholtens, Sylvain Sebert, Verena Sengpiel, Marcus A Tuke, Marc Vaudel, Michael N Weedon, Gonneke Willemsen, Andrew R Wood, Hanieh Yaghootkar, Louis J Muglia, Meike Bartels, Caroline L Relton, Craig E Pennell, Leda Chatzi, Xavier Estivill, John W Holloway, Dorret I Boomsma, Grant W Montgomery, Joanne M Murabito, Tim D Spector, Christine Power, Marjo-Ritta Järvelin, Hans Bisgaard, Struan F A Grant, Thorkild I A Sørensen, Vincent W Jaddoe, Bo Jacobsson, Mads Melbye, Mark I McCarthy, Andrew T Hattersley, M Geoffrey Hayes, Timothy M Frayling, Marie-France Hivert, Janine F Felix, Elina Hyppönen, William L Lowe, David M Evans, Debbie A Lawlor, Bjarke Feenstra, Rachel M Freathy

**Affiliations:** 1 Institute of Biomedical and Clinical Science, University of Exeter Medical School, University of Exeter, Royal Devon and Exeter Hospital, Exeter EX2 5DW, UK,; 2 Translational Research Institute, University of Queensland Diamantina Institute, Brisbane, QLD, Australia,; 3 Centre for Environmental and Preventive Medicine, Wolfson Institute of Preventive Medicine, Barts and the London School of Medicine and Dentistry, Queen Mary University of London, London, UK,; 4 European Centre for Environment and Human Health, University of Exeter, The Knowledge Spa, Truro TR1 3HD, UK,; 5 Department of Preventive Medicine, Northwestern University Feinberg School of Medicine, Chicago, IL, USA,; 6 Oxford Centre for Diabetes, Endocrinology and Metabolism, University of Oxford, Oxford, UK,; 7 Wellcome Trust Centre for Human Genetics, University of Oxford, Oxford, UK,; 8 Department of Epidemiology Research, Statens Serum Institut, Copenhagen, Denmark,; 9 Division of Epidemiology, Department of Genes and Environment, Norwegian Institute of Public Health, Oslo, Norway,; 10 Medical Research Council Integrative Epidemiology Unit at the University of Bristol, Oakfield House, Oakfield Grove, Bristol BS8 2BN, UK,; 11 Population Health Science, Bristol Medical School, University of Bristol, Oakfield House, Oakfield Grove, Bristol BS8 2BN, UK,; 12 The Generation R Study Group, Erasmus MC, University Medical Center Rotterdam, 3000 CA Rotterdam, The Netherlands,; 13 Center for Applied Genomics, The Children’s Hospital of Philadelphia, Philadelphia, PA, USA,; 14 Copenhagen Prospective Studies on Asthma in Childhood (COPSAC), Faculty of Health Sciences, University of Copenhagen, Copenhagen, Denmark,; 15 Danish Pediatric Asthma Center, Copenhagen University Hospital, Gentofte, Denmark; 16 Institute of Health Sciences, University of Oulu, Oulu, Finland,; 17 Department of Twin Research, King's College London, St. Thomas' Hospital, London, UK,; 18 Department of Biostatistics, Boston University School of Public Health, Boston, MA, USA,; 19 Framingham Heart Study, Framingham, MA, USA,; 20 QIMR Berghofer Medical Research Institute, Royal Brisbane Hospital, Herston, QLD 4029, Australia,; 21 EMGO Institute for Health and Care Research, VU University Medical Center, Amsterdam, The Netherlands,; 22 Department of Biological Psychology, Vrije Universiteit Amsterdam, 1081 BT Amsterdam, The Netherlands,; 23 Centre de Recherche du Centre Hospitalier Universitaire de Sherbrooke, Sherbrooke, QC, Canada,; 24 Medical Research Council Lifecourse Epidemiology Unit, University of Southampton, Southampton, UK,; 25 Pompeu Fabra University (UPF), Barcelona, Spain,; 26 IMIM (Hospital del Mar Medical Research Institute), Barcelona, Spain,; 27 ISGlobal, Centre for Research in Environmental Epidemiology (CREAL), Barcelona, Spain,; 28 Division of Obstetrics and Gynaecology, The University of Western Australia, Perth, Australia,; 29 Institute of Genetic Medicine, Newcastle University, Central Parkway, Newcastle upon Tyne NE1 3BZ, UK,; 30 Human Genetics Division, Cincinnati Children’s Hospital Medical Center, Cincinnati, OH, USA,; 31 Center for Prevention of Preterm Birth, Perinatal Institute, Cincinnati Children's Hospital Medical Center, OH, USA,; 32 March of Dimes Prematurity Research Center Ohio Collaborative, Cincinnati, OH, USA,; 33 Population, Policy and Practice, UCL Great Ormond Street Institute of Child Health, University College London, London, UK,; 34 ECOGENE-21 and Lipid Clinic, Chicoutimi Hospital, Saguenay, QC, Canada,; 35 Department of Biochemistry, Université de Sherbrooke, Sherbrooke, QC, Canada,; 36 Division of Human Genetics, The Children’s Hospital of Philadelphia, Philadelphia, PA, USA,; 37 Department of Pediatrics, Perelman School of Medicine, University of Pennsylvania, Philadelphia, PA, USA,; 38 FIMM Institute for Molecular Medicine Finland, Helsinki University, Helsinki FI-00014, Finland,; 39 Department of Clinical Science, KG Jebsen Center for Diabetes Research, University of Bergen, Bergen, Norway,; 40 Department of Pediatrics, Haukeland University Hospital, Bergen 5021, Norway,; 41 Department of Genetics and Bioinformatics, Domain of Health Data and Digitalisation, Institute of Public Health, Oslo, Norway,; 42 The First Affiliated Hospital of Jinan University, Guangzhou 510630, China,; 43 Institute of Nutritional Science, University of Potsdam, Potsdam, Germany,; 44 Department of Epidemiology, Erasmus MC, University Medical Center Rotterdam, 3000 CA Rotterdam, The Netherlands,; 45 NIHR Southampton Biomedical Research Centre, University of Southampton and University Hospital Southampton NHS Foundation Trust, Southampton, UK,; 46 Genetic Section, Department of Life Sciences and Biotechnology, University of Ferrara, Ferrara, Italy,; 47 Department of Pediatrics, University of Iowa, Iowa City, IA, USA,; 48 Research Unit of Obstetrics & Gynecology, Institute of Clinical Research, University of Southern Denmark, Odense, Denmark,; 49 Center for Cardiovascular Research, Charité, Berlin, Germany,; 50 Subdirección de Salud Pública y Adicciones de Gipuzkoa, Donostia/San Sebastián, Spain,; 51 Instituto de Investigación Sanitaria BIODONOSTIA, Donostia/San Sebastián, Spain,; 52 Department of Epidemiology and Biostatistics, School of Public Health, Medical Research Council-Health Protection Agency Centre for Environment and Health, Faculty of Medicine, Imperial College London, London, UK,; 53 Department of Obstetrics and Gynecology, Sahlgrenska Academy, Sahgrenska University Hospital, Gothenburg, Sweden,; 54 Department of Social Medicine, University of Crete, Crete, Greece,; 55 Human Development & Health, Faculty of Medicine, University of Southampton, Southampton, UK,; 56 Section of General Internal Medicine, Boston University School of Medicine, Boston, MA, USA,; 57 Biocenter Oulu, University of Oulu, Oulu, Finland,; 58 Unit of Primary Care, Oulu University Hospital, FI-90220 Oulu, 90029 OYS, Finland,; 59 Department of Children and Young People and Families, National Institute for Health and Welfare, FI-90101 Oulu, Finland,; 60 Department of Public Health, Faculty of Health and Medical Sciences, University of Copenhagen, Copenhagen, Denmark,; 61 Novo Nordisk Foundation Center for Basic Metabolic Research, Faculty of Health and Medical Sciences, University of Copenhagen, Copenhagen, Denmark,; 62 Department of Pediatrics, Erasmus MC, University Medical Center Rotterdam, 3000 CA Rotterdam, The Netherlands,; 63 Department of Medicine, Stanford University School of Medicine, Stanford, CA, USA,; 64 Oxford National Institute for Health Research (NIHR) Biomedical Research Centre, Churchill Hospital, Oxford, UK,; 65 Division of Endocrinology, Metabolism and Molecular Medicine, Northwestern University Feinberg School of Medicine, Chicago, IL, USA,; 66 Department of Population Medicine, Harvard Pilgrim Health Care Institute, Harvard Medical School, Boston, MA, USA,; 67 Diabetes Center, Massachussetts General Hospital, Boston, MA, USA,; 68 Department of Medicine, Universite de Sherbrooke, QC, Canada,; 69 Centre for School of Population Health Research, School of Health Sciences, and Sansom Institute, University of South Australia, Adelaide, Australia; 70 South Australian Health and Medical Research Institute, Adelaide, Australia

## Abstract

Genome-wide association studies of birth weight have focused on fetal genetics, whereas relatively little is known about the role of maternal genetic variation. We aimed to identify maternal genetic variants associated with birth weight that could highlight potentially relevant maternal determinants of fetal growth. We meta-analysed data on up to 8.7 million SNPs in up to 86 577 women of European descent from the Early Growth Genetics (EGG) Consortium and the UK Biobank. We used structural equation modelling (SEM) and analyses of mother–child pairs to quantify the separate maternal and fetal genetic effects. Maternal SNPs at 10 loci (*MTNR1B*, *HMGA2*, *SH2B3*, *KCNAB1*, *L3MBTL3*, *GCK*, *EBF1*, *TCF7L2*, *ACTL9*, *CYP3A7*) were associated with offspring birth weight at *P *<* *5 × 10^−8^. In SEM analyses, at least 7 of the 10 associations were consistent with effects of the maternal genotype acting via the intrauterine environment, rather than via effects of shared alleles with the fetus. Variants, or correlated proxies, at many of the loci had been previously associated with adult traits, including fasting glucose (*MTNR1B*, *GCK* and *TCF7L2*) and sex hormone levels (*CYP3A7*), and one (*EBF1*) with gestational duration. The identified associations indicate that genetic effects on maternal glucose, cytochrome P450 activity and gestational duration, and potentially on maternal blood pressure and immune function, are relevant for fetal growth. Further characterization of these associations in mechanistic and causal analyses will enhance understanding of the potentially modifiable maternal determinants of fetal growth, with the goal of reducing the morbidity and mortality associated with low and high birth weights.

## Introduction

Individuals with birth weights approaching the lower or upper ends of the population distribution are more at risk of adverse neonatal and later-life health outcomes and mortality than those of average weight (1–5). The factors influencing birth weight involve both maternal and fetal genetic contributions in addition to the environment. Genome-wide association studies (GWASs) testing for common variant effects on *own* birth weight (‘fetal’ GWAS) have so far identified 60 robustly associated loci (6–8). The influence of common maternal genetic variation on offspring birth weight, beyond the effects of transmitted genetic variation, is poorly understood. Studies estimating the variance in birth weight explained by fetal or maternal genetic factors, using data on twins (9,10), families (11) or mother–child pairs with genome-wide common variant data (8,12), have consistently estimated a distinct maternal genetic contribution, which is smaller than the fetal genetic contribution, with estimates ranging from 3% to 22% of the variance explained (relative to 24% to 69% for fetal genetics).

**Figure 1.**
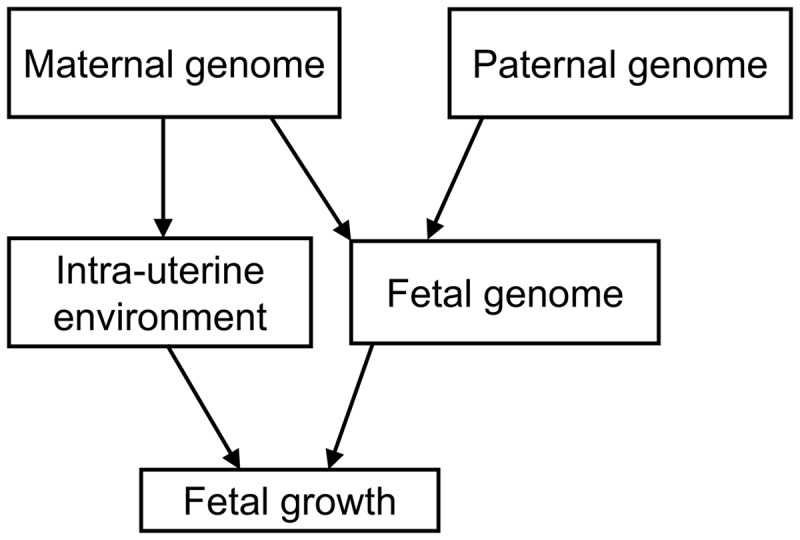
A schematic diagram illustrating that maternal genetic factors may influence fetal growth indirectly through the intra-uterine environment, or directly through inheritance by the fetus.

Maternal genotypes may influence key maternal phenotypes, such as circulating levels of glucose and other metabolic factors, which could cross the placenta and affect the growth of the fetus. For example, women with hyperglycemia due to rare heterozygous mutations in the *GCK* gene have babies who are heavier at birth (provided the babies do not inherit the mutation) due to intrauterine exposure to high maternal glucose levels (13). Additionally, maternal genotypes may act upon other maternal attributes, such as vascular function or placental transfer of nutrients, which are also likely to influence fetal growth. Such maternal environmental effects could in turn influence fetal growth separately from the effects of any growth-related genetic variants that are inherited by the fetus directly from the mother (Fig. 1). Supporting evidence for such effects from analyses of common genetic variants includes positive associations between maternal weighted allele scores for body mass index (BMI) or fasting glucose and offspring birth weight, and an inverse association between a maternal weighted allele score for systolic blood pressure and offspring birth weight (14).

The goal of the current study was to apply a GWAS approach to identify maternal genetic variants associated with offspring birth weight. This could potentially highlight novel pathways by which the maternal genotype influences offspring birth weight through the intra-uterine environment. We performed a meta-analysis of GWASs of offspring birth weight using maternal genotypes in up to 86 577 women of European descent from 25 studies, including 37 945 participants from studies collaborating in the Early Growth Genetics (EGG) Consortium and 48 632 participants from the UK Biobank (Supplementary Material, Fig. S1). We identified 10 loci, and showed, using a novel structural equation model and analyses in mother–child pairs, that the majority of these were maternal effects that were independent of the fetal genotype.

## Results

The basic characteristics of study participants in the EGG Consortium discovery, EGG follow-up and UK Biobank GWAS analyses are presented in Supplementary Material, Tables S1–S3, respectively.

### Maternal SNPs at 10 loci were associated with offspring birth weight at *P* < 5 × 10^−8^

**Figure 2.**
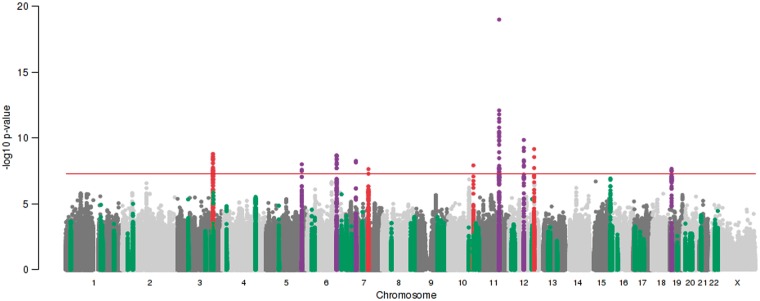
Manhattan plot of associations between 8 723 755 maternal autosomal SNPs and 17 352 maternal X-chromosome SNPs (all MAF >1%) and offspring birth weight from the meta-analysis of up to 86 577 women. SNP position across the chromosomes (*x*-axis) and results of association tests between maternal genotype and offspring birth weight adjusted for sex and, where available, gestational duration (–log_10_*P*-value; *y*-axis) are shown. The index maternal SNP from the current study and all SNPs within 500 kb of that SNP are highlighted either in red, or in purple. Those in purple indicate loci at which the index maternal SNP from the current study was within 500k of an index SNP associated previously with own birth weight (i.e. in a ‘fetal GWAS of birth weight’) at *P* < 5 **×** 10^−8^ (8). SNPs within 500 kb of the 54 other index SNPs previously identified in the fetal GWAS of birth weight are highlighted in green. The red, horizontal line indicates a *P* value of 5 **×** 10^−8^.

We identified 10 autosomal loci that were associated with offspring birth weight at *P *< 5 **×** 10^−8^ (Fig. 2, Table 1, Supplementary Material, Figs S2 and S3 and Table S4). The linkage disequilibrium (LD) score regression intercept (15) from the overall meta-analysis was 1.009, so there was little change in the test statistics after adjusting for this inflation. Three of these loci (*KCNAB1*, *EBF1* and *CYP3A7*) were identified in UK Biobank data only, and the index SNPs were unavailable in the EGG Consortium data. Consideration of results for proxy SNPs at these three loci from the EGG meta-analysis is in the next section. For the index SNPs at the other seven loci, we observed no strong evidence of heterogeneity in allelic effects between the EGG Consortium and UK Biobank components of the meta-analysis (Supplementary Material, Fig. S4 and Table S4). The majority of the index SNPs mapped to non-coding sequence and were not in strong LD with any coding variants (*r*^2^ < 0.95), but the index SNP in *SH2B3*, rs3184504, is a non-synonymous coding variant (R262W). Approximate conditional analysis (see Materials and Methods) showed no evidence of secondary signals at any locus at *P* < 5 **×** 10^−8^. In combination, the 10 loci explained 1.4% [standard error (SE) = 1.2%] of variance in birth weight, whereas the variance in birth weight captured by all autosomal genotyped variants on the UK Biobank array was considerably greater: 11.1% (SE = 0.6%).

**Table 1.** Ten maternal genetic loci associated with offspring birth weight (*P* < 5 **×** 10^−8^) in a European ancestry meta-analysis of up to 86 577 women

*Locus*	Lead SNP	Chr	Position (bp, b37)	Alleles	EAF	EGG + UKBB meta-analysis		
				Effect/Other		Effect (SE)	*P*-value	*n*
*MTNR1B*	rs10830963	11	92708710	G/C	0.29	0.052 (0.006)	1.0 **×** 10^−19^	71 341
*HMGA2*	rs1351394	12	66351826	T/C	0.49	0.034 (0.005)	1.4 **×** 10^−10^	68 247
*SH2B3*	rs3184504	12	111884608	C/T	0.52	0.033 (0.005)	6.9 **×** 10^−10^	68 249
*KCNAB1*^a^	rs7629460	3	155829938	C/A	0.59	0.039 (0.007)	1.6 **×** 10^−9^	48 632
*KCNAB1*^b^	rs9872556	3	155829855	T/C	0.57	0.029 (0.005)	8.1 **×** 10^−8^	68 253
*L3MBTL3*	rs9375694	6	130356608	G/A	0.31	0.035 (0.006)	2.1 **×** 10^−9^	68 223
*GCK*	rs2971669	7	44231778	T/C	0.23	0.038 (0.007)	5.5 **×** 10^−9^	68 162
*EBF1*^a^	rs12520982	5	157894747	T/C	0.74	0.041 (0.007)	9.9 **×** 10^−9^	48 632
*EBF1*^b^	rs2964484	5	157897437	A/G	0.71	0.031 (0.006)	4.6 **×** 10^−7^	67 547
*TCF7L2*	rs7903146	10	114758349	T/C	0.30	0.034 (0.006)	1.2 **×** 10^−8^	68 253
*ACTL9*	rs2918299	19	8787273	C/T	0.83	0.041 (0.007)	2.2 **×** 10^−8^	67 603
*CYP3A7*^a^	rs45446698	7	99332948	G/T	0.04	0.089 (0.016)	2.4 **×** 10^−8^	48 632

Effects were aligned to the birth weight-raising allele and are presented in SD units [1 SD of birth weight = 484 g (7)]. Chr, chromosome; bp, base pair; b37, build 37; EAF, effect allele frequency.

a Index SNPs available in UK Biobank only.

b Results for proxy SNPs (*r*^2^ = 1) at *KCNAB1* and *EBF1* available in both UK Biobank and EGG (imputed to HapMap Phase 2) are shown below the index SNPs, with full details in Supplementary Material, Table S4 (in both cases there was weak evidence of heterogeneity of effect size between EGG and UK Biobank, *P* < 0.01). No proxy for rs45446698 at *CYP3A7* with *r*^2^ > 0.5 was available in HapMap Phase 2.

### Birth weight-raising alleles at *KCNAB1* and *EBF1* were associated with longer gestational duration

The associations at *KCNAB1*, *EBF1* and *CYP3A7* resulted from analysis of UK Biobank data only, and index SNPs were unavailable in the EGG Consortium meta-analysis (imputed to HapMap Phase 2). To investigate further the evidence for association at these loci, we identified proxy SNPs (*r*^2^ = 1) for *KCNAB1* (rs9872556) and *EBF1* (rs2964484) that were available in HapMap Phase 2 (no proxy SNP was available at *r*^2^ > 0.5 at the *CYP3A7* locus). Meta-analysis of the EGG Consortium and UK Biobank data showed weaker evidence of association overall, with some evidence of heterogeneity between the EGG meta-analysis and UK Biobank (*P* = 0.008 and 0.007, respectively; Table 1, Supplementary Material, Table S4 and Fig. S4). In the UK Biobank, women reported the birth weight of their first child, but not the duration of gestation. In contrast, analyses of birth weight in all but one EGG study [Queensland Institute of Medical Research (QIMR), *n* = 892] were adjusted for the duration of gestation. It is therefore possible that the associations observed with birth weight at *KCNAB1* and *EBF1* in the UK Biobank reflect primary associations with gestational duration. Look-ups of the index SNPs and HapMap 2 proxy SNPs in a published dataset of the top 10 000 associated SNPs from a GWAS of gestational duration and preterm birth in 43 568 women (16) showed evidence of association at both *EBF1* (*P* < 10^−12^) and *KCNAB1* (*P* < 10^−3^; Supplementary Material, Table S5). The birth weight-raising alleles were associated with longer gestational duration.

### Five associated SNPs were independent of those identified in previous fetal GWAS of birth weight

The index SNPs at four of the identified loci (*SH2B3*, *KCNAB1*, *TCF7L2* and *CYP3A7*), mapped >2 Mb away from, and were statistically independent of any index SNPs previously associated with birth weight at *P* < 5x10^−8^ in a fetal GWAS (*r*^2^ < 0.05) (8). A summary of candidate genes at these four loci is presented in Supplementary Material, Table S6 [corresponding information for the other loci was reported in (8)]. At *MTNR1B* and *HMGA2*, the same index SNP was associated with birth weight in the same direction in both the current study and the previous fetal GWAS. At the four remaining loci, the maternal GWAS index SNPs were within 0.5 to 15 kb of previously reported fetal GWAS index SNPs with very different strengths of pairwise LD between the maternal and fetal GWAS index SNPs. At the *EBF1* and *ACTL9* loci, the maternal and fetal GWAS index SNPs were in strong LD (*r*^2^ = 0.95 and 0.99, respectively), and the directions of association were consistent, suggesting that they were tagging the same causal variant. At the *L3MBTL3* locus, the maternal and fetal directions of association were consistent, but the index SNPs were weakly correlated (*r*^2^ = 0.13), and conditional analyses in UK Biobank suggested weakening of the association when the *L3MBTL3* SNP identified in the fetal GWAS was accounted for [unconditional effect (SE) = 0.033 (0.007), *P* *=* 1.4 **×** 10^−6^; conditional effect (SE) =0.024 (0.008), *P* *=* 1.2 **×** 10^−3^; Supplementary Material, Table S7]. At the *GCK* locus, the minor allele frequencies of the maternal and fetal GWAS index SNPs were very different (0.23 and 0.009, respectively), and in low pairwise LD (*r*^2^ = 0.002). Analysis conditional on the fetal GWAS index SNP in UK Biobank did not alter the association at the maternal index SNP (Supplementary Material, Table S7), suggesting that at *GCK*, the maternal association with birth weight was distinct from the previously reported fetal association.

### Structural equation modelling applied to UK Biobank data suggested most associations were driven by the maternal genotype

**Figure 3.**
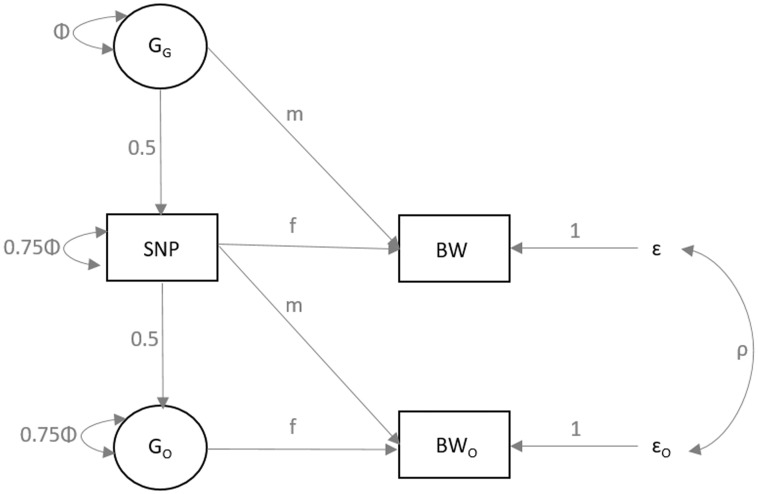
Diagram of the SEM used to estimate the conditional fetal and maternal effects on birth weight for each of the genome-wide significant SNPs. The three observed variables (in rectangles) are the birth weight of the individual (BW), the birth weight of their offspring (BW_O_) and the genotype of the individual (SNP). The latent variables (in circles) are the genotype of the individual’s mother (G_G_) and the genotype of the individual’s first offspring (G_O_). The total variance of the latent genotypes for the individual’s mother (G_G_) and offspring (G_O_) and for the observed SNP variable is set to Φ [i.e. variance (G_G_) = Φ; variance (SNP) = 0.25Φ + 0.75Φ; variance(G_O_) = 0.25 Φ + 0.75Φ]. The *m* and *f* path coefficients refer to maternal and fetal effects, respectively. The residual error terms for the birth weight of the individual and their offspring are represented by *ɛ* and *ɛ*_O_, respectively, and we estimate the variance of both of these terms in the SEM. The covariance between residual genetic and environmental sources of variation is given by *ρ*.

**Figure 4.**
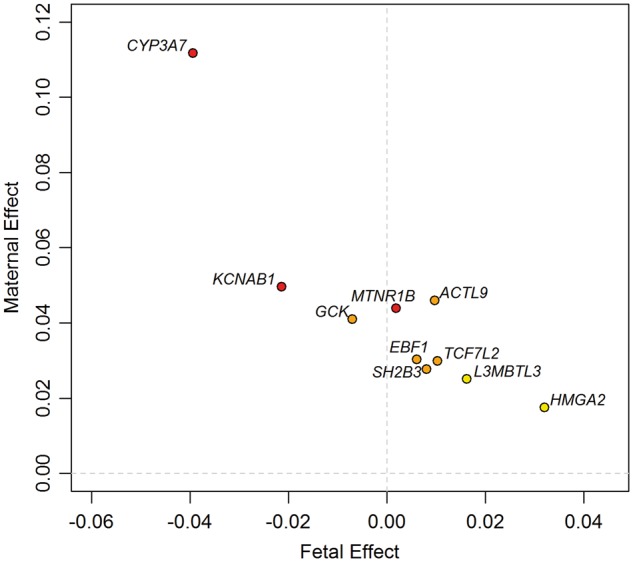
Independent maternal and fetal effects on birth weight at the 10 identified loci, estimated from a meta-analysis of results using an SEM in unrelated UK Biobank participants with results using conditional analysis in maternal–fetal pairs. All SNPs are aligned to the birth weight-raising allele reported in Table 1. The colour of each dot indicates the maternal genetic association *P*-value for birth weight, adjusted for the fetal genetic association: red, *P* < 0.0001; orange, 0.0001 ≤ *P* < 0.001; yellow, 0.001 ≤ *P* < 0.05.

The partial overlap between associations identified in the current study and those identified in the previous fetal GWAS of birth weight (Fig. 2 and Supplementary Material, Fig. S2) illustrates the expected correlation between maternal and fetal genotypes (*r* ≈ 0.5). The associations between maternal genotype and birth weight identified here may represent indirect effects of maternal genotype on birth weight acting via the maternal intrauterine environment, or primary effects of the fetal genotype on birth weight that are captured (due to correlation) when assaying the maternal genotype, or a mixture of maternal and fetal effects. Analysis of UK Biobank data using structural equation modelling (SEM; *n* = 78 674 male and female unrelated participants, of whom 33 238 individuals only reported their own birth weight, 20 963 women only reported the birth weight of their first child and 24 473 women reported their own birth weight and that of their first child; see Materials and Methods and Fig. 3) provided estimates of maternal effects adjusted for fetal genotype, and vice versa, and suggested that the associations at the majority of the loci were driven by the maternal genotype (Fig. 4 and Supplementary Material, Table S8). In particular, the adjusted maternal effects estimated at seven of the loci (*MTNR1B*, *KCNAB1*, *GCK*, *EBF1*, *TCF7L2*, *ACTL9* and *CYP3A7*) were separated from the adjusted fetal effect estimates by at least 2 SEs. The only locus at which the point estimate for the adjusted fetal effect was larger than that of adjusted maternal effect was *HMGA2*, suggesting this association was driven by the fetal genotype*.* Additional analyses (i) adjusting for fetal genotype in up to 8705 mother–child pairs and (ii) comparing the unadjusted maternal effect estimates from the overall maternal GWAS (*n* = up to 86 577) with those from a published fetal GWAS (*n* = 143 677), provided supporting evidence that the majority of the effects were maternally driven (Supplementary Material, Fig. S5 and Tables S8 and S9).

### Known associations at the identified loci highlighted potentially relevant maternal traits including fasting glucose, blood pressure, immune function and sex hormone levels

Look-ups of index SNPs (*n* = 7 loci), or SNPs in close LD (*n* = 2 loci at *r*^2^ > 0.9; *n* = 1 at *r*^2^ = 0.4), in available GWAS datasets for cardiometabolic and growth-related traits revealed several associations at *P* *<* 5 × 10^−8^ (Supplementary Material, Table S10), and further information on previously reported associations was obtained from the NHGRI-EBI catalog of GWAS (see Materials and Methods).

The maternal birth weight-associated variants at *MTNR1B*, *GCK* and *TCF7L2* loci are known to be associated with fasting glucose and Type 2 diabetes susceptibility (17,18), with the glucose-raising allele associated with higher offspring birth weight.

The C-allele of the missense variant, rs3184504, in *SH2B3*, associated with higher birth weight in our study, has been associated with multiple cardiovascular traits [lower SBP and DBP (19,20), altered lipid levels and lower risk of coronary artery disease, CAD (21,22)], altered haematological traits (23–26), lower risk of autoimmune diseases and autoimmune disorders (27–32), lower kynurenine levels (33), higher risk of tonsillectomy (34) and higher risk of colorectal and endometrial cancer (35).

The maternal birth weight-raising allele at *ACTL9* was in LD (*r*^2^ = 1) with alleles of nearby variants associated with lower risk of atopic dermatitis (36) and higher risk of tonsillectomy (34).

At the *CYP3A7* locus, an SNP in LD (rs34670419, *r*^2^ = 0.74) with our identified variant, rs45446698, has been associated with levels of the hormones, progesterone and dehydroepiandrosterone sulphate (DHEAS) (37). The maternal birth weight-raising allele was associated with lower hormone levels.

The index SNP, rs12520982, at *EBF1* is in LD (*r*^2^ = 1) with rs2963463, recently associated with gestational duration and preterm birth (16). SNPs in weak LD (*r*^2^ < 0.1) with *EBF1* rs12520982 were previously associated with blood pressure traits (19,38,39), and rs12520982 showed moderate evidence of association with SBP (*P* = 1 **×** 10^−5^) and DBP (*P* = 1 **×** 10^−3^) in the UK Biobank data (Supplementary Material, Table S10). There were no known prior associations with the birth weight-associated variation at *KCNAB1*.

The variants at *HMGA2* and *L3MBTL3* have been associated with adult height (40). At *HMGA2*, and possibly also at *L3MBTL3*, the association with birth weight is through the fetal allele, not the maternal allele, so these associations with adult height are relevant for offspring, not mother. Associations at *HMGA2* were additionally observed with other growth and development phenotypes: infant length (41), infant head circumference (42) and primary or permanent tooth eruption (43,44).

To identify biological pathways underlying maternal regulation of birth weight, we performed gene-set enrichment analysis using Meta-Analysis Gene-set EnrichmeNT of variant Associations (MAGENTA) (45). Seven pathways reached false discovery rate (FDR) < 0.05, including three involved in the metabolism of xenobiotics (Supplementary Material, Table S11).

## Discussion

In this study, we have identified variants in the maternal genome at 10 loci that are robustly associated with offspring birth weight. Five of the identified associations are independent of those reported in previous fetal GWAS of birth weight (8), bringing the total of known independent common variant associations with birth weight to 65. Because maternal and fetal genotype are correlated (*r* = 0.5), loci identified in GWAS of birth weight to date could either represent effects of the maternal genotype, acting via the intrauterine environment, or direct effects of the fetal genotype, or a mixture of the two (Fig. 1). Our analyses, and those of 58 previously reported loci (46), suggest that although the majority of the 65 known associations indicate direct effects of the fetal genotype, at least 7 associations from the current study [those at *MTNR1B*, *EBF1*, *ACTL9*, *KCNAB1*, *GCK*, *TCF7L2* and *CYP3A7*, of which the first 3 were initially identified in fetal GWAS (8)] indicate maternal intrauterine effects.

The index SNP, rs45446698, at the *CYP3A7* locus, is an expression quantitative trait locus (eQTL) for *CYP3A7* in adrenal gland tissue (47). The *CYP3A7* gene is part of the cytochrome P450 family 3 subfamily A gene cluster, which encodes enzymes responsible for the metabolism of multiple and diverse endogenous and exogenous molecules (48), and SNP rs45446698 tags a haplotype of seven highly correlated variants in the *CYP3A7* promoter, known as the CYP3A7*1C allele (49,50). The *CYP3A7* gene is predominantly expressed in fetal development, but CYP3A7*1C results in expression in adult carriers (50,51). The CYP3A7*1C allele, and correlated SNPs, have been associated with circulating levels of DHEAS, progesterone and 2-hydroxylation pathway estrogen metabolites (37,52,53). There were no associations between offspring birth weight and maternal SNPs at each of nine loci (independent of *CYP3A7*) that are also known to influence levels of DHEAS or progesterone (37,54) (data not shown), suggesting that neither DHEAS nor progesterone levels *per se* are likely to explain the association with birth weight. Because CYP3A enzymes metabolize a diverse range of substrates, there are many possible mechanisms by which maternal CYP3A7*1C might be associated with birth weight. In our conditional analysis, we observed weak evidence of an independent association with the fetal allele at this locus in the opposite direction to that of the maternal allele. Further analyses in larger samples will be required to confirm this and to investigate possible mechanisms underlying this association. However, the association at this locus, together with the results of the gene-set enrichment analysis, which highlighted pathways involved in xenobiotic metabolism, suggests that it is a key avenue for future research into fetal outcomes.

The birth weight-raising maternal alleles at the identified loci (*MTNR1B*, *GCK* and *TCF7L2*) are strongly associated with higher fasting glucose and Type 2 diabetes in non-pregnant adults (17,18), and with glycemic traits and gestational diabetes mellitus in pregnant women (55–57). The association between raised maternal glucose and higher offspring birth weight is the result of higher fetal insulin secretion in response to increased placental transfer of glucose (58). Our results confirm previous maternal candidate gene associations with birth weight at *TCF7L2* and *GCK* (55,59,60) and demonstrate the key role of maternal glucose levels in influencing offspring birth weight (14,61). Notably, the Type 2 diabetes risk allele at each of these three loci was not associated with birth weight independently of the maternal allele when present in the fetus. This is contrary to what has been seen at other Type 2 diabetes loci such as *ADCY5* and *CDKAL1*, where risk alleles in the fetus were associated with lower birth weight (8). However, there is an additional low-frequency fetal variant at the *GCK* locus, which is independent of the glucose-raising maternal variant associated with higher birth weight in the current study (8). Taken together with the known effects on birth weight of both maternal and fetal rare heterozygous *GCK* mutations (13), a complex picture of allelic variation relevant to fetal growth is emerging at this locus.

The association with birth weight at *HMGA2* was previously identified in a fetal GWAS of birth weight (same index SNP) (8), and our analyses showed that the maternal SNP in our study was probably capturing a direct effect of the SNP in the fetus on skeletal growth, given previous associations with infant length, head circumference and adult height (40–42). The *L3MBTL3* locus identified in our study is also a known height locus, and the associated variant was correlated (*r*^2^ = 0.13) with an SNP associated with birth weight in the previous fetal GWAS (8). It was less clear from our analyses whether the association at *L3MBTL3* originated from the maternal or fetal genotype. However, analyses of maternal height alleles transmitted to offspring versus those not transmitted to offspring suggest that the majority of the association between maternal height and offspring birth weight are due to direct effects of fetal inherited alleles (62).

Our exploration of known associations at the remaining four loci indicated a number of potentially relevant maternal traits that could influence birth weight via the intrauterine environment, including higher blood pressure (associations at *SH2B3* and suggestive associations at *EBF1*, both between the blood pressure raising maternal allele and lower offspring birth weight), which has been causally associated with lower birth weight in Mendelian randomization analyses (14), and immune function (associations at *SH2B3* and *ACTL9*). However, further studies are needed to elucidate the mechanisms at these loci and at *KCNAB1*, which showed no previous associations with other traits.

We observed weak evidence of heterogeneity of effect sizes between the EGG Consortium and UK Biobank components of our meta-analysis at the *KCNAB1* and *EBF1* loci, which led us to investigate possible explanations. A key difference was that birth weight was adjusted for duration of gestation in the majority of EGG studies, whereas the duration of gestation was unavailable in the UK Biobank. This raised the possibility that birth weight associations at *KCNAB1* and *EBF1* might arise from a primary effect on gestational duration, i.e. these loci could be primarily influencing the timing of delivery, rather than fetal growth. It is of course possible that the heterogeneity indicated false positive associations in the UK Biobank dataset that were not replicated in the EGG dataset. However, directionally consistent evidence of association with gestational duration and preterm birth in a recently published GWAS (*P* < 5 **×** 10^−8^ at *EBF1*; *P* < 10^−3^ at *KCNAB1*) suggests that this is unlikely.

There were some limitations to our study. First, the birth weight of first child was self-reported by mothers in the UK Biobank study, and so was likely subject to more error variation and potential bias than measured birth weight. However, maternal reports of offspring birth weight have been shown to be accurate (63,64), and we showed that the birth weight of first child variable was associated with maternal smoking, height, BMI and socio-economic position in the expected directions. A second limitation of our study was that by performing a maternal GWAS of birth weight that does not account for the fetal genotype, the analysis was biased against identifying loci at which the fetal genotype exerts opposing effects. Proof-of-principle that such loci exist is demonstrated by the effects on birth weight of rare mutations in the *GCK* gene, which act in opposite directions when present in either mother or fetus, but result in normal birth weight if both mother and fetus inherit the mutation (13). Our analysis conditional on fetal genotype at the 10 loci using a novel method (46) had greatly increased power to resolve maternal versus fetal effects compared with previous analyses in limited numbers of mother–child pairs (8). Although it is not yet computationally feasible to run such an analysis genome-wide, future studies will benefit from considering maternal and fetal genotype simultaneously at the discovery stage and are thereby likely to uncover further loci.

In conclusion, we have identified 10 maternal genetic loci associated with offspring birth weight, 5 of which were not previously identified in fetal GWAS of birth weight, and at least 7 of which represent maternal intrauterine effects. Collectively, the identified associations highlight key roles for maternal glucose and cytochrome P450 activity and potential roles for maternal blood pressure and immune function. Future genetic, mechanistic and causal analyses will be required to characterize such intrauterine effects, leading to greater understanding of the maternal determinants of fetal growth, with the goal of reducing the morbidity and mortality associated with low and high birth weights.

## Materials and Methods

### EGG Consortium discovery studies: genotyping, imputation and GWAS analysis

We studied 19 626 unrelated women of European ancestry from 11 studies with maternal genome-wide genotypes and offspring birth weight available. These included two sub-samples from the 1958 British birth cohort [1958BC-WTCCC2 (65), *n* = 836; 1958BC-T1DGC (27), *n* = 858]; the Avon Longitudinal Study of Parents and Children (ALSPAC, *n* = 7304) (66); a sub-sample of the Danish National Birth Cohort from the Genetics of Extreme Overweight in Young Adults study (DNBC-GOYA, *n* = 1805) (67); population-based controls from a case–control study of pre-term birth in the DNBC (DNBC-PTBCTRLS, *n* = 1656) (68); the Hyperglycemia and Adverse Pregnancy Outcome study (HAPO, *n* = 1280) (56); the Norwegian Mother and Child cohort study (MoBa, *n* = 650) (69); the Northern Finland 1966 Birth Cohort study (NFBC1966, *n* = 2035) (70); the Netherlands Twin Register (*n* = 707) (71); the QIMR study of adult twins (*n* = 892) (72); the Twins UK study (TwinsUK, *n* = 1603) (73).

Genotypes in each study were obtained through high-density SNP arrays and up to ∼2.5 million autosomal SNPs were imputed to HapMap Phase II. Study protocol was approved at each study centre by the local ethics committee and written informed consent had been obtained from all participants and/or their parent(s) or legal guardians. Study descriptions and basic characteristics of samples in the discovery phase are presented in Supplementary Material, Table S1.

Within each study, we converted offspring birth weight (BW, g) to a *z*-score [(BW value – mean(BW))/standard deviation(BW)] to allow comparison of data across studies. We excluded multiple births, stillbirths, congenital anomalies (where known) and births before 37 weeks of gestation (where known). We assessed the association between each SNP and offspring birth weight using linear regression of the birth weight *z*-score against maternal genotype (additive genetic model), with sex and gestational duration as covariables (gestational duration was unavailable in the QIMR study, which contributed 4.5% of EGG participants). Ancestry principal components were included as covariables where necessary in the individual studies. Genome-wide association analyses were conducted using PLINK (74), SNPTEST (75), Mach2qtl (76) or Beagle (77) (see Supplementary Material, Table S1).

### Genome-wide meta-analysis of 11 EGG Consortium discovery studies

Before meta-analysis, SNPs with a minor allele frequency (MAF) < 0.01 and poorly imputed SNPs [info < 0.8 (PLINK), r2hat < 0.3 (MACH or Beagle) or proper_info < 0.4 (SNPTEST)] were excluded. To adjust for inflation in test statistics generated in each cohort, genomic control (78) was applied once to each individual study (see Supplementary Material, Table S1 for *λ* values in each study). Data annotation, exchange and storage were facilitated by the SIMBioMS platform (79). Quality control of individual study results and fixed-effects inverse variance meta-analyses were undertaken by two meta-analysts in parallel at different study centres using the software package METAL (2009–10-10 release) (80). We obtained association statistics for a total of 2 422 657 SNPs in the meta-analysis for which at least 7 of the 11 studies were included. The genomic control inflation factor, *λ*, in the overall meta-analysis was 1.007.

### Follow-up of 18 SNPs in 13 additional EGG Consortium studies

We selected 15 SNPs that surpassed a *P*-value threshold of *P* < 1 **×** 10^−5^ for follow-up in additional, independent studies. Of these, one SNP (rs11020124) was in LD (*r*^2^ = 0.63, 1000 Genomes Pilot 1 data) with SNP rs10830963 at the *MTNR1B* locus known to be associated with fasting glucose and Type 2 diabetes (81). We assumed that these represented the same association signal. Given its robust association with maternal glycemic traits likely to impact on offspring birth weight, we took only rs10830963 forward for follow-up at this locus. We identified three further SNPs at loci with robust evidence (*P* < 5 **×** 10^−8^) of association with other phenotypes, and therefore higher prior odds of association with birth weight: rs2971669 near *GCK* (*r*^2^ = 0.73 with rs4607517 associated with fasting glucose) (60); rs204928 in *LMO1* (*r*^2^ = 0.90 with rs110419 associated with neuroblastoma) (82) and rs7972086 in *RAD51AP1* (*r*^2^ = 0.27 with rs2970818 associated with serum phosphorus concentration) (83). We took forward SNPs rs4607517, rs204928 and rs7972086 for follow-up at these loci, giving a total of 18 SNPs to be examined in additional studies.

The descriptions, genotyping details and basic phenotypic characteristics of the follow-up studies are presented in Supplementary Material, Table S2. Of a total of 13 follow up studies (*n* = 18 319 individuals), 9 studies (*n* = 15 288) provided custom genotyping of between 4 and 18 SNPs, whereas 4 studies (*n* = 3031 individuals) had *in silico* genome-wide or exome-wide SNP genotypes available. Where SNPs were imputed, we included only those with quality scores (r2hat or proper_info) >0.8. We excluded directly genotyped SNPs showing evidence of deviation from Hardy–Weinberg Equilibrium at *P* < 0.0028 (Bonferroni corrected for 18 tests). Where genotypes were unavailable for the index SNP, we used *r*^2^ > 0.8 proxies (see Supplementary Material, Table S12).

### Preparation, quality control and genetic analysis in UK Biobank samples

UK Biobank data were available for 502 655 participants, of whom 273 463 were women (84), and of these women, 216 811 reported the birth weight of their first child (in pounds) either at the baseline or follow-up assessment visit. We converted pounds to kg (multiplying by 0.45) for use in our analyses. No information was available on gestational duration or offspring sex. A total of *n* = 64 072 women with offspring birth weight data available also had genotype data available in the May 2015 data release. Women identified as not of British descent (*n* = 9681) were excluded from the analysis along with those reporting offspring birth weights of <2.5 or > 4.5 kg (*n* = 5479). ‘British descent’ was defined as individuals who both self-identified as white British and were confirmed as ancestrally Caucasian using principal components analyses (http://biobank.ctsu.ox.ac.uk; date last accessed August 2, 2017). A total of 1976 of the women were asked to repeat the questionnaire at a follow-up assessment and therefore had two reports of birth weight of first child. Those with values differing by ≥1 lb (0.45 kg) were excluded (*n* = 280). This resulted in *n* = 48 632 women with both genotype data and a valid offspring birth weight value, which was *z*-score transformed for analysis (Supplementary Material, Table S3). UK Biobank carried out stringent quality control of the GWAS genotype scaffold prior to imputation up to a reference panel of a combined 1000 Genomes Project Consortium and UK10K Project Consortium. We tested for association with birth weight of first child using a linear mixed model implemented in BOLT-LMM (85) to account for cryptic population structure and relatedness. Genotyping array was included as a binary covariate in the regression model. Total chip heritability (i.e. the variance explained by all autosomal polymorphic genotyped SNPs passing quality control) was calculated using restricted maximum likelihood implemented in BOLT-LMM (85). We additionally analysed the association between birth weight of first child and directly genotyped SNPs on the X chromosome in 45 445 unrelated women identified by UK Biobank as white British. We excluded SNPs with evidence of deviation from Hardy–Weinberg equilibrium (*P* < 1 **×** 10^−6^), MAF < 0.01 or overall missing rate > 0.015, resulting in 17 352 SNPs for analysis in PLINK v.1.07, with the first 5 ancestry principal components as covariates.

In both the full UK Biobank sample and our refined sample, birth weight of first child was associated with mother’s smoking status, maternal BMI and maternal height in the expected directions (Supplementary Material, Table S3). 

### Overall meta-analysis of discovery and follow-up samples

A flowchart of the overall study design is presented in Supplementary Material, Figure S1. We performed inverse variance, fixed-effects meta-analysis of the association between each SNP and birth weight *z*-score in up to 25 discovery and follow-up studies combined (maximum total *n* = 86 577 women; 8 723 755 SNPs with MAF ≥ 0.01 plus 17 352 X-chromosome SNPs in 45 445 women) using METAL (80). To check for population substructure or relatedness that was not adequately accounted for in the analysis, we examined the intercept value from univariate LD score regression (15).

### Approximate conditional analysis

At each of the identified loci, we looked for the presence of multiple distinct association signals in the region 1 Mb up- and down-stream from the lead SNP through approximate conditional analysis. Conditional and joint analysis in the analysis program, genome-wide complex trait analysis (86) was applied to identify secondary signals that attained genome-wide significance (*P* < 5 **×** 10^−8^) using a sample of 10 000 individuals selected at random from the UK Biobank to approximate patterns of LD between variants in these regions.

### Candidate gene search

To search for candidate genes at the four loci not already covered by the previous fetal GWAS of birth weight (8), we identified the nearest gene, searched PubMed for relevant information on genes within 300 kb of the index SNP, and queried the index SNP for eQTL or proxy SNPs (*r*^2^ > 0.8) reported from GTEx v4, GEUVADIS, and 11 other studies using Haploreg v4.1 (http://archive.broadinstitute.org/mammals/haploreg/haploreg.php; date last accessed August 2, 2017).

### Estimating maternal and fetal genetic effects at the identified loci

Because of the small number of cohorts with both maternal and offspring genotype data available to conduct conditional analysis, we developed a novel method using SEM to estimate the conditional maternal and fetal genetic effects on birth weight, which we subsequently applied to the maternal and offspring birth weight data in the UK Biobank. SEM is a flexible multivariable statistical approach that allows investigators to model the covariance between an observed set of variables (i.e. here an individual’s genotype, their birth weight and their offspring’s birth weight) as a function of several latent unobserved variables (i.e. here the genotype of the individual’s mother and the genotype of their offspring). The full details of the SEM method for estimating the conditional fetal and maternal effects are described elsewhere (46). Briefly, as seen in Figure 3, we fitted a structural equation model to three observed variables from the UK Biobank study; the participant’s own self-reported birth weight, the birth weight of the first child reported by the women and the genotype of the participants. Our model included two latent variables; one for the individual’s mother (i.e. grand-maternal genotype) and one for the genotype of the participant’s offspring. We know these latent variables are correlated on average 50% with the individual’s own genotype, hence the path coefficient between each of the latent variables and the observed genotype was set to 0.5. Our model also included residual error terms for the participant’s own birth weight and the birth weight of their first child, a covariance parameter to quantify similarity between the error terms, and a variance parameter to model variation in the observed genotype. Using this model, we were able to simultaneously estimate the effect of maternal and fetal genotypes on offspring birth weight.

To fit the SEM, we used OpenMx (87) in R (version 3.3.2) (88) with the raw UK Biobank data, and the *P*-value for the fetal and maternal paths was calculated using a Wald test. We fitted a second SEM without the child and maternal path to conduct a 2 degree of freedom test for the effect of the SNP on birth weight.

Genotype data from the UK Biobank May 2015 release was used for analysis. We included 57 711 participants who reported their own birth weight and 45 436 women who reported the birth weight of their first child, giving a total of 78 674 unique individuals in the analysis (24 473 women had both their own and their offspring’s birth weight). Individuals who were not of ‘British descent’ (as defined earlier), or were related to others in the sample, or who were part of multiple births, were excluded. The birth weight of offspring phenotype was prepared as described earlier, whereas own birth weight was prepared as described previously (8). The included sample was smaller than that used previously to fit the same structural equation model to a different set of SNPs in the UK Biobank (46), because of a narrower definition of ethnicity and a slightly narrower offspring birth weight range. The narrower definitions were chosen here to match closely the sample analysed in the main GWAS of the current study. We adjusted the individuals’ own birth weight for sex, and both birth weight measures for the 12 genetically determined principal components and genotyping batch before creating *z*-scores for analysis.

We analysed up to 8705 mother–child pairs from 4 studies with both maternal and fetal genotypes available [ALSPAC, Exeter Family Study of Childhood Health, HAPO (non-GWAS) and DNBC-PTBCTRLS]. We used linear regression to test the association between birth weight *z*-score and maternal genotype conditional on fetal genotype and vice versa (also adjusting analyses for sex and gestational duration). We combined the results from the individual studies using inverse variance meta-analysis with fixed effects. We performed a further meta-analysis to combine the overall estimates with those from the SEM using UK Biobank data.

### Look-ups in published GWAS and NHGRI GWAS catalog

We looked up associations between the 10 identified loci and various anthropometric and cardiometabolic traits in available GWAS result sets. The traits and sources are presented in Supplementary Material, Table S10. Where the index SNPs at *KCNAB1*, *EBF1* and *CYP3A7* were unavailable, we used proxies (*r*^2^ = 0.99, 1.00 and 0.41, respectively). Because GWAS summary statistics for blood pressure were not publicly available, we used the UK Biobank May 2015 genetic data release and tested associations between the SNPs and systolic and diastolic blood pressure (SBP and DBP) in 127 968 and 127 776 British descent participants, respectively. Two blood pressure readings were taken approximately 5 min apart using an automated Omron blood pressure monitor. Two valid measurements were available for most participants, and the average was taken. Individuals were excluded if the two readings differed by more than 4.56 SD (1 SD was equal to 19.7 and 13.1 mmHg for SBP and DBP, respectively), and blood pressure measurements more than 4.56 SD away from the mean were excluded. We accounted for blood pressure medication use by adding 15 mmHg to the SBP measure and 10 mmHg to the DBP measure in those reporting regular use of any antihypertensive. Blood pressure was adjusted for age, sex and centre location and then inverse normalized before analysis.

We additionally queried the NHGRI-EBI catalog of published GWAS (http://www.ebi.ac.uk/gwas/home, last accessed 2 August 2017) for associations *P* < 5 **×** 10^−8^ between any additional traits or diseases and SNPs within 500 kb of, and in LD with, the index SNP at each locus.

### Gene set enrichment analysis

We used MAGENTA to test for pathway-based associations using summary statistics from the overall meta-analysis (45). The software mapped each gene to the SNP with the lowest *P* value within a 110 kb upstream and 40 kb downstream window. The *P* value (representing a gene score) was corrected for confounding factors such as gene size, SNP density and LD-related properties in a regression model. Genes within the HLA-region were excluded. Genes were then ranked by their adjusted gene scores. The observed number of gene scores in a given pathway with a ranked score above a given threshold (95th and 75^th^ percentiles) was calculated and this statistic was compared with 1 000 000 randomly permuted pathways of the same size. This generated an empirical *P* value for each pathway, and we considered pathways reaching FDR < 0.05 to be of interest. The 3230 biological pathways tested were from the BIOCARTA, Gene Ontology, Ingenuity, KEGG, PANTHER and REACTOME databases, with a small number of additional custom pathway.

## Supplementary Material

Supplementary Material is available at *HMG* online. Summary statistics from the meta-analysis are available at http://egg-consortium.org/.

## Supplementary Material

Supplementary Information

Supplementary Tables
