# Supplementary material for: Genome-wide association study of offspring birth weight in 86 577 women identifies five novel loci and highlights maternal genetic effects that are independent of fetal genetics
Source: Hum Mol Genet. 2018 Jan 3;27(4):742–56. doi: 10.1093/hmg/ddx429 (PMC5886200; doi:10.1093/hmg/ddx429)
Supplement: Supplementary Information [file sup-info-ddx429.docx]

**SUPPLEMENTARY MATERIAL**

**Genome-wide association study of offspring birth weight in 86,577 women identifies five novel loci and highlights maternal genetic effects that are independent of fetal genetics**

| **Content** | **Page** |
| --- | --- |
| **Figure S1.** Flowchart of study design | 2 |
| **Figure S2.** Quantile-quantile plot of 8,723,755 maternal autosomal SNPs and 17,352 maternal X-chromosome SNPs (all MAF >1%) from the meta-analysis of up to 86,577 women | 3 |
| **Figure S3.** Regional plots of association between maternal genotype and offspring birth weight at 10 loci | 4 |
| **Figure S4.** Forest plots showing associations between the index SNP at the identified loci and birth weight in all available studies | 6 |
| **Figure S5.** Comparison of maternal effect sizes with fetal effect sizes at 10 loci identified in the current study | 8 |
| **Table S3.** Basic characteristics of UK Biobank participants, with observational associations between reported birth weight of first child and other variables | 9 |
| **Table S4.** Associations between SNPs at 10 loci and birth weight in different stages of the study | 10 |
| **Table S5** Associations between SNPs at *KCNAB1* and *EBF1* and gestational duration or preterm birth in publicly available data from a GWAS of 43,568 women (1). | 12 |
| **Table S6.** Candidate genes at newly identified loci | 13 |
| **Table S7.** Analysis of index SNPs from maternal GWAS of birth weight conditional on index SNPs from fetal GWAS of birth weight (2) at the at *L3MBTL3* and *GCK* loci | 14 |
| **Table S8.** Associations between maternal genotype and birth weight, adjusted for fetal genoype, and vice versa | 15 |
| **Table S9.** Look-up of fetal genotype associations with birth weight (unadjusted for maternal genotype) in up to 143,675 individuals, for the 10 loci identified in the maternal GWAS of birth weight (unadjusted for fetal genotype) | 17 |
| **Table S11.** Results of gene set enrichment analysis | 18 |
| **Table S12.** Details of proxy SNPs analysed in EGG follow-up studies if the index SNP was unavailable | 19 |
| **References** | 20 |
| **Study-specific acknowledgements** | 21 |
| **Details of funding by study** | 23 |
| **EGG Consortium membership** | 28 |

**Large Supplementary Tables provided in separate Excel spreadsheets:**

**Table S1:** Basic characteristics, genotyping, imputation and analysis details for participants in the EGG Discovery studies

**Table S2:** Basic characteristics, genotyping, imputation and analysis details for participants in the EGG Follow-up studies

**Table S10:** Look-ups of 10 birth weight-associated SNPs in available GWAS data for other growth-related and cardiometabolic traits

**Figure S1.** Flowchart of study design.

**
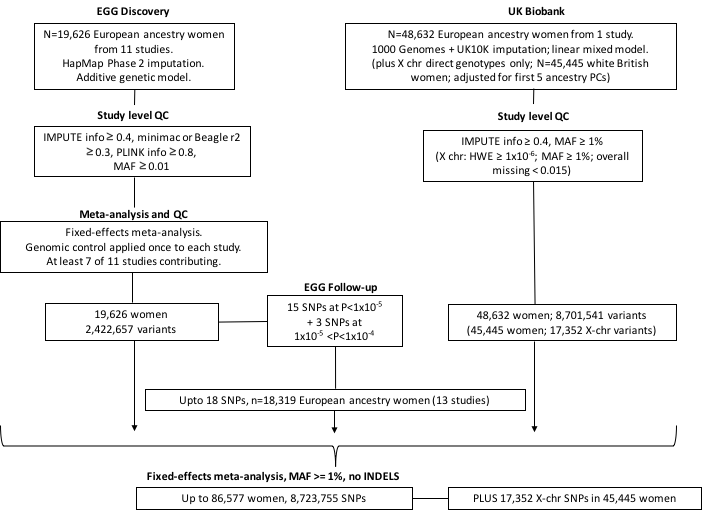
**

**Figure S2.** Quantile-quantile plot of 8,723,755 maternal autosomal SNPs and 17,352 maternal X-chromosome SNPs (all MAF >1%) from the meta-analysis of up to 86,577 women. Observed versus expected –log_10_ *P*-values are plotted in black for all SNPs, and in pink excluding 60 index SNPs (+/-500kb) identified in a previous GWAS of own birth weight (“fetal GWAS”). The fine black lines represent the expected –log_10_ *P*-values under the null distribution and the 95% concentration bands, which are an approximation to the 95% confidence intervals around the expected line.


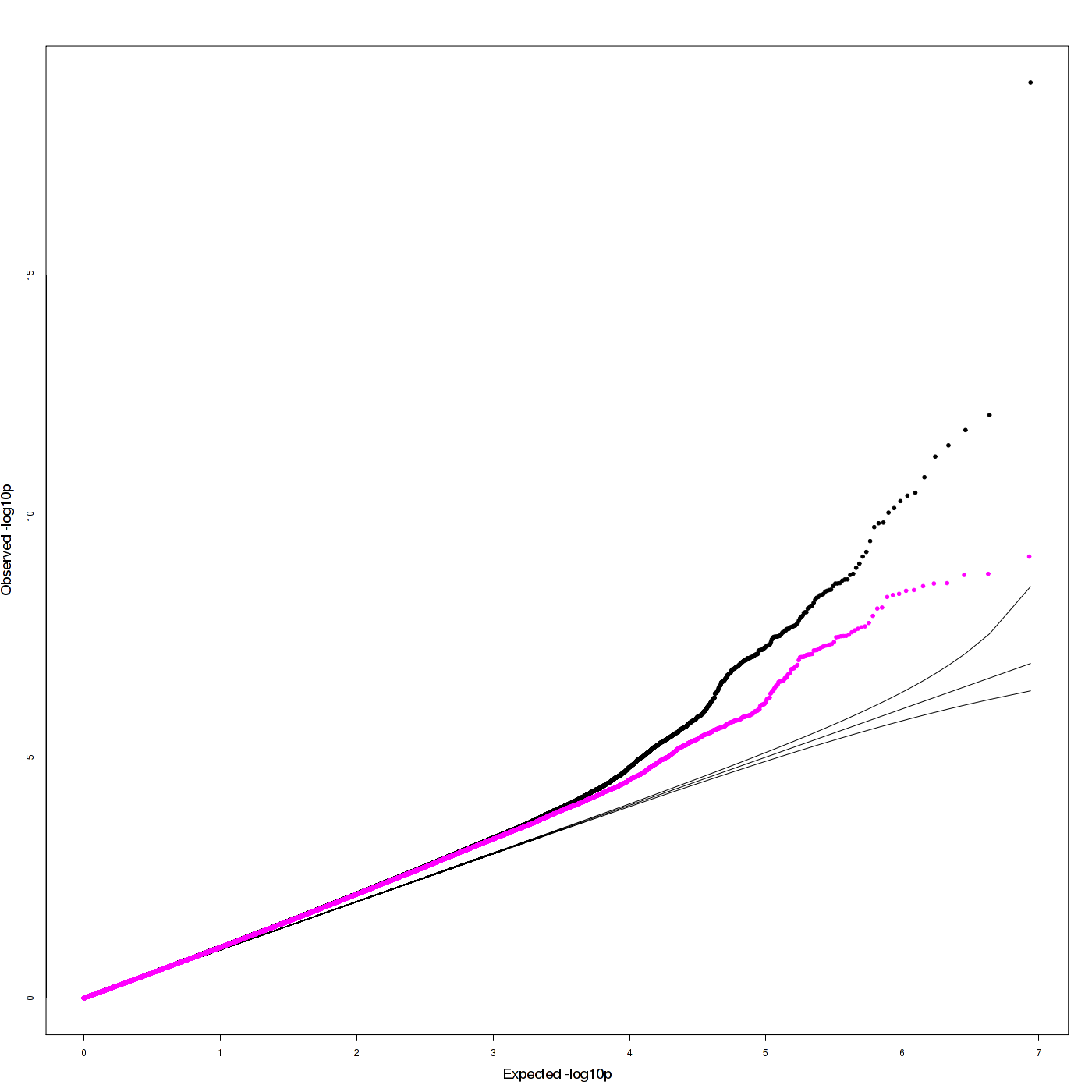


**Figure S3.** Regional plots of association between maternal genotype and offspring birth weight at 10 loci for which associations at index SNPs (purple diamonds) reached *P* < 5x10^-8^.

**
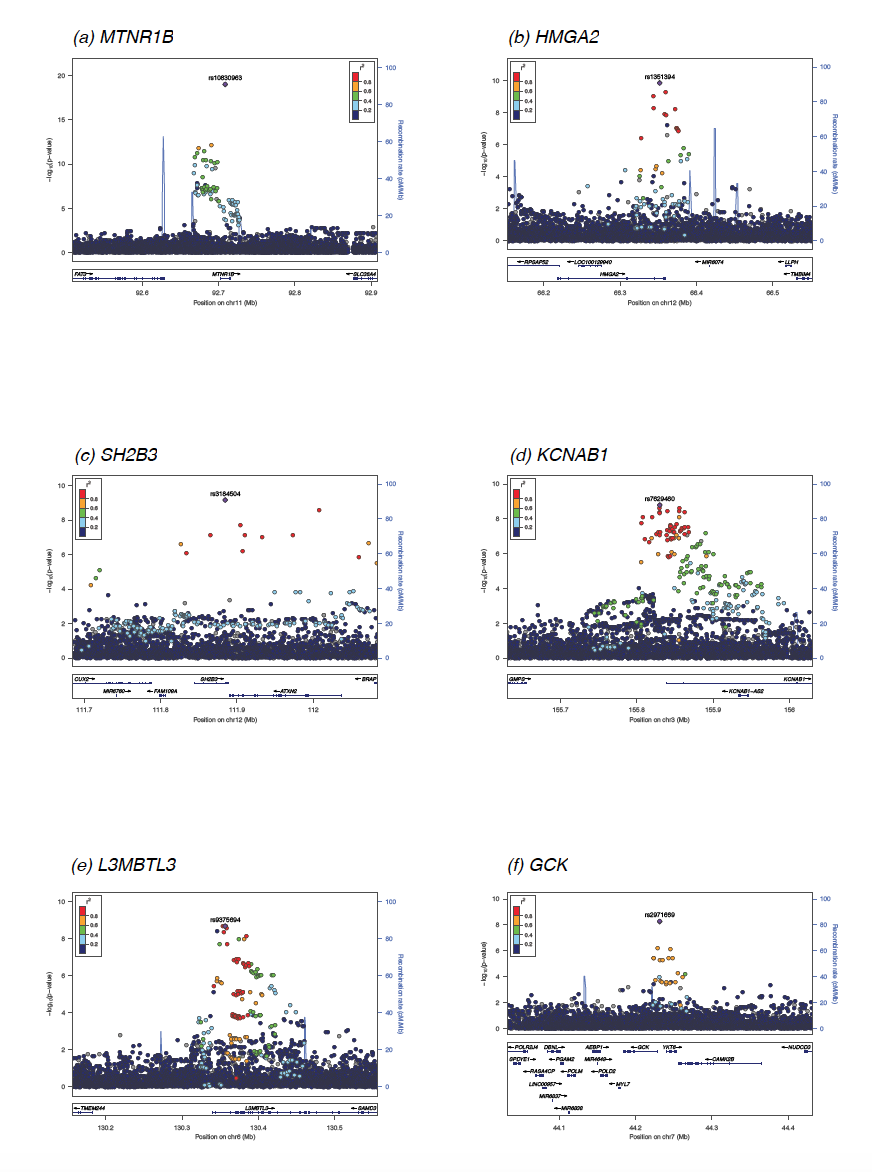
**

**
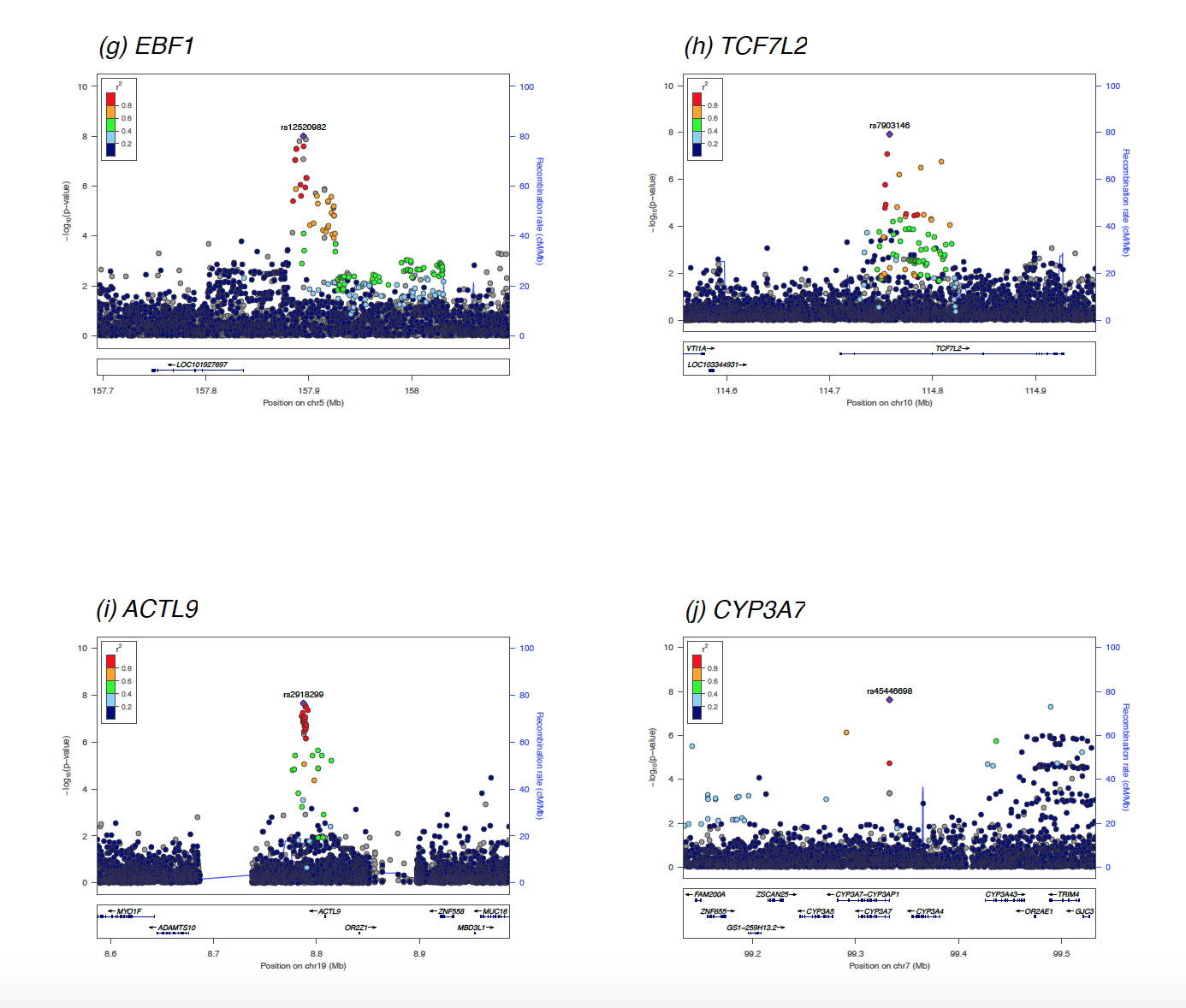
**

**Figure S4.** Forest plots showing associations between the index SNP at the identified loci and birth weight in all available studies. At *KCNAB1* and *EBF1* index SNP was only available in UK Biobank, so the plots show results for the most closely correlated proxy (r^2^ = 1) that was also available in the EGG meta-analysis (the result for the index SNP is also marked as “UKBiobank Lead Signal”. There is no plot for *CYP3A7* because the lead SNP was only available in UK Biobank and there were no proxies at r^2^ >0.5 available in the EGG meta-analysis.

1. *MTNR1B* (b) *HMGA2*


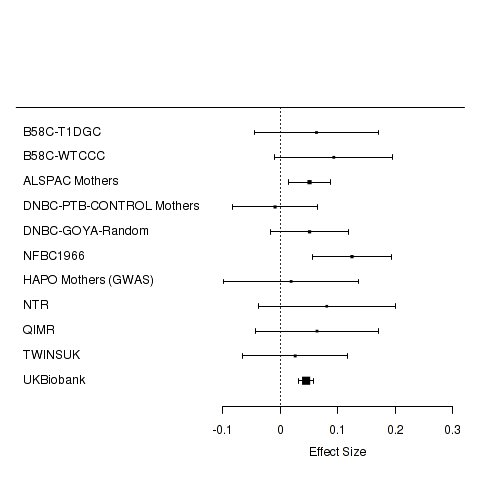

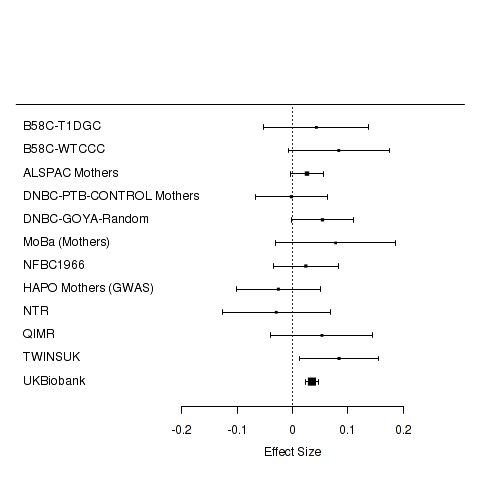


(c) *SH2B3* (d) *KCNAB1*

*
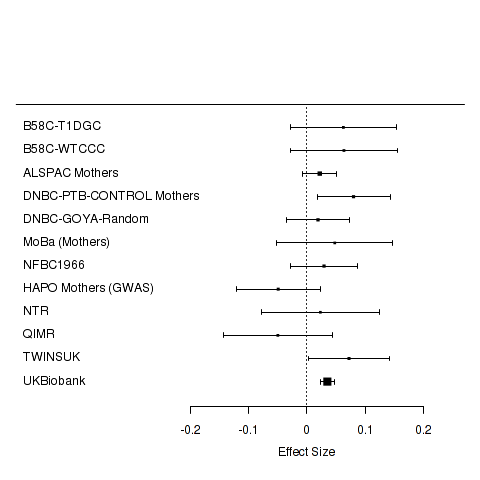

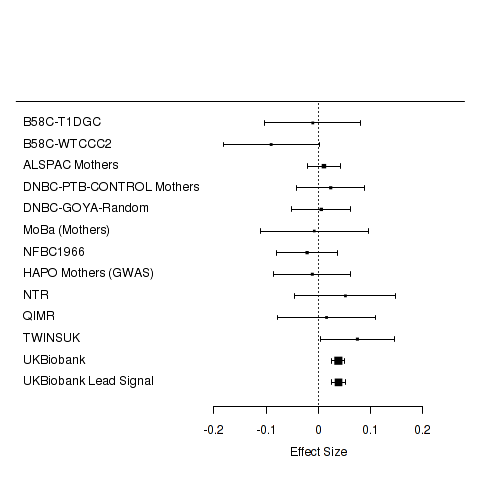
*

(e) *L3MBTL3* (f) *GCK*

*
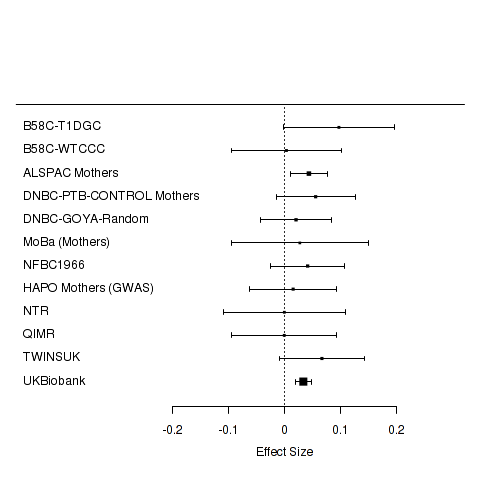

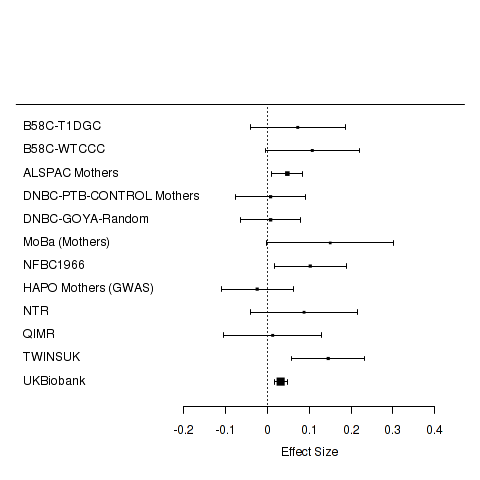
*

(g) *EBF1* (h) *TCF7L2*

*
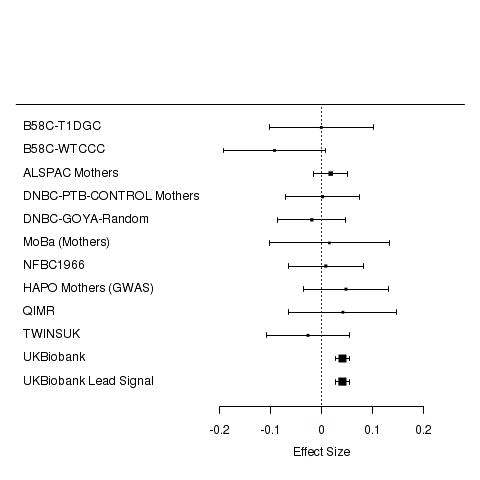

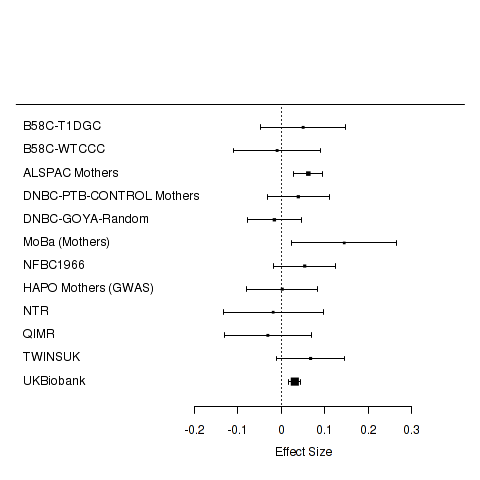
*

(h) *ACTL9*

*
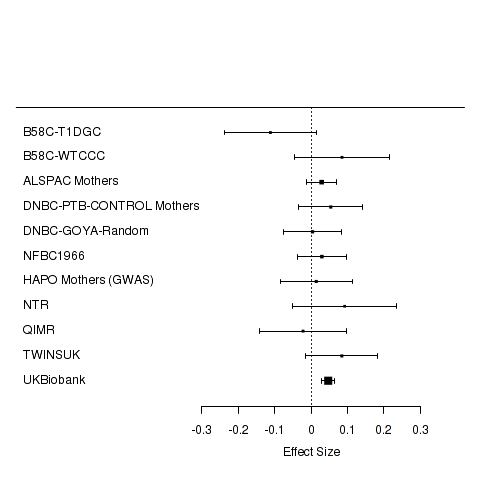
*

**Figure S5.** Comparison of maternal effect sizes with fetal effect sizes at 10 loci identified in the current study. For each locus, the following effect sizes (with 95% CI) are shown, all aligned to the same allele: Maternal_GWAS, maternal allelic effect on offspring BW (from meta-analysis of n = up to 86,577 European mothers); Maternal_unadjusted, maternal allelic effect on offspring birth weight (unconditioned, n = up to 8705 mother–child pairs); Maternal_adjusted, maternal effect (conditioned on fetal genotype, n = up to 8705); Fetal_GWAS, fetal allelic effect on BW (from European ancestry meta-analysis of n =up to 143,677 individuals (2)); Fetal_unadjusted, fetal allelic effect on BW (unconditioned in n = up to 8705); Fetal_adjusted, fetal effect (conditioned on maternal genotype, n = up to 8705).

**
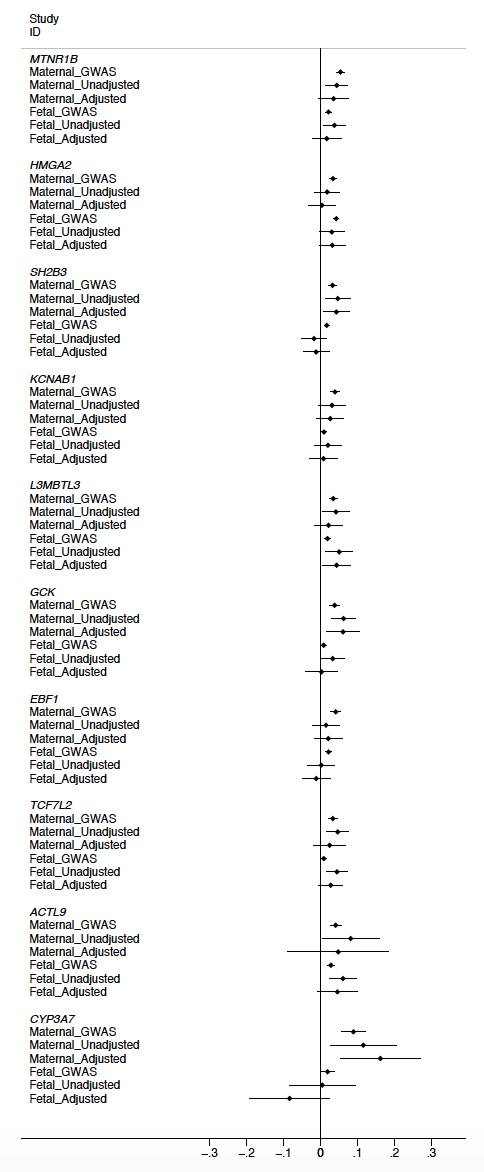
**

**Table S3.** Basic characteristics of UK Biobank participants, with observational associations between reported birth weight of first child and other variables

| Ethnicity | | British descent |
| --- | --- | --- |
| Country (Sample source) | | UK |
| Year(s) of birth of offspring | | 1934 to 2008 |
| Number of mothers included in the analyais | | 48632 |
| Birth weight data | | Reported by mothers at baseline or follow-up study visit |
| Birth weight of first child [Mean (sd)], grams (converted from pounds) | | 3255 (396) |
| Gestational age available? | | No |
| Offspring sex available? | | No |
| Maternal age at baseline [Mean (sd)], years | | 57.2 (7.7) |
| Maternal age at first birth [Mean (sd)], years | | 25.9 (5.1) |
| Maternal BMI at baseline [Mean (sd)], kg/m2 | | 27.43 (4.80) |
| Mean (SD) birth weight of first child by smoking information collected at baseline (n=214,367) | Never (59.6 %) | 3198 (569) |
|  | Former (32.3 %) | 3194 (568) |
|  | Current (8.1 %) | 3101 (587) |
|  | P value for association | 4.9 x 10^-57^ |
| Mean (SD) birth weight of first child by maternal BMI tertile (in kg/m^2^) measured at baseline (n=215,657) | 1 (12.12 to 25.03) | 3170 (559) |
|  | 2 (25.04 to 28.68) | 3195 (567) |
|  | 3 (28.68 to 74.68) | 3206 (590) |
|  | P value for association | 5.0 x 10^-46^ |
| Mean (SD) birth weight of first child by maternal height tertile (in cm) measured at baseline (n=216,096) | 1 (75 to 164) | 3131 (563) |
|  | 2 (164 to 173) | 3280 (569) |
|  | 3 (173 to 209) | 3402 (585) |
|  | P value for association | <1 x 10^-100^ |
| Mean (SD) birth weight of first child by tertiles of Townsend deprivation index at baseline (n=216,547) | 1 (-6.26 to 3.15) | 3214 (559) |
|  | 2 (-3.14 to -0.59) | 3201 (563) |
|  | 3 (-0.58 to 11) | 3145 (592) |
|  | P value for association | <1 x 10^-100^ |

**Table S4.** Associations between SNPs at 10 loci and birth weight in different stages of the study.

| ***Locus*** | **Lead SNP** | **Chr** | **Position (bp, b37)** | **Alleles** | **EAF** | **EGG discovery meta-analysis** | | | | **EGG replication** | | | | **UK Biobank only (*N*=48632)** | | **EGG + UK Biobank meta-analysis** | | | |
| --- | --- | --- | --- | --- | --- | --- | --- | --- | --- | --- | --- | --- | --- | --- | --- | --- | --- | --- | --- |
|  |  |  |  | **Effect / Other** |  | **Effect (SE)** | ***P*-value** | ***N* [*N* studies]** | **Het *P-*value** | **Effect** | ***P*-value** | ***N* [*N* studies]** | **Het *P-*value** | **Effect (SE)** | ***P*-value** | **Effect (SE)** | ***P*-value** | ***N*** | **Het *P-*value** |
|  |  |  |  |  |  |  |  |  |  |  |  |  |  |  |  |  |  |  |  |
| *MTNR1B** | rs10830963 | 11 | 92708710 | G/C | 0.29 | 0.055 (0.012) | 3.8 x 10^-6^ | 18971 [10] | 0.36 | 0.071 (0.011) | 1.6 x 10^-10^ | 16851 [11] | 0.30 | 0.045 (0.007) | 1.9 x 10^-10^ | 0.052 (0.006) | 1.0 x 10^-19^ | 71341 | 0.27 |
| *HMGA2* | rs1351394 | 12 | 66351826 | T/C | 0.49 | 0.031 (0.010) | 1.2 x 10^-3^ | 19615 [11] | 0.45 | - | - | - | - | 0.035 (0.006) | 3.1 x 10^-8^ | 0.034 (0.005) | 1.4 x 10^-10^ | 68247 | 0.70 |
| *SH2B3* | rs3184504 | 12 | 111884608 | C/T | 0.52 | 0.028 (0.009) | 3.2 x 10^-3^ | 19617 [11] | 0.24 | - | - | - | - | 0.035 (0.006) | 5.1 x 10^-8^ | 0.033 (0.005) | 6.9 x 10^-10^ | 68249 | 0.54 |
| *KCNAB1* | rs7629460 | 3 | 155829938 | C/A | 0.59 | - | - | - | - | - | - | - | - | 0.039 (0.007) | 1.6 x 10^-9^ | - | - | - | - |
| *KCNAB1†* | rs9872556 | 3 | 155829855 | T/C | 0.57 | 0.007 (0.010) | 0.45 | 19620 [11] | 0.40 | - | - | - | - | 0.039 (0.007) | 2.9 x 10^-9^ | 0.029 (0.005) | 8.1 x 10^-8^ | 68253 | 0.008 |
| *L3MBTL3* | rs9375694 | 6 | 130356608 | G/A | 0.31 | 0.038 (0.011) | 3.6 x 10^-4^ | 19591 [11] | 0.92 | - | - | - | - | 0.033 (0.007) | 1.4 x 10^-6^ | 0.035 (0.006) | 2.1 x 10^-9^ | 68223 | 0.74 |
| *GCK* | rs2971669 | 7 | 44231778 | T/C | 0.23 | 0.053 (0.012) | 1.5 x 10^-5^ | 19530 [11] | 0.14 | - | - | - | - | 0.032 (0.008) | 3.2 x 10^-5^ | 0.038 (0.007) | 5.5 x 10^-9^ | 68162 | 0.16 |
| *GCK‡* | rs4607517 | 7 | 44235668 | A/G | 0.21 | 0.035 (0.013) | 5.9 x 10^-3^ | 19619 [11] | 0.01 | 0.046 (0.012) | 2.7 x 10^-4^ | 18128 [13] | 0.10 | 0.030 (0.008) | 2.7 x 10^-4^ | 0.035 (0.006) | 7.2 x 10^-9^ | 86379 | 0.14 |
| *EBF1* | rs12520982 | 5 | 157894747 | T/C | 0.74 | - | - | - | - | - | - | - | - | 0.041 (0.007) | 9.9 x 10^-9^ | - | - | - | - |
| *EBF1†* | rs2964484 | 5 | 157897437 | A/G | 0.71 | 0.006 (0.011) | 0.62 | 18914 [10] | 0.66 | - | - | - | - | 0.041 (0007) | 1.2 x 10^-8^ | 0.031 (0.006) | 4.6 x 10^-7^ | 67547 | 0.007 |
| *TCF7L2* | rs7903146 | 10 | 114758349 | T/C | 0.30 | 0.039 (0.011) | 2.7 x 10^-4^ | 19621 [11] | 0.23 | - | - | - | - | 0.031 (0.007) | 9.5 x 10^-6^ | 0.034 (0.006) | 1.2 x 10^-8^ | 68253 | 0.54 |
| *ACTL9* | rs2918299 | 19 | 8787273 | C/T | 0.83 | 0.028 (0.013) | 3.6 x 10^-2^ | 18971 [10] | 0.46 | - | - | - | - | 0.047 (0.009) | 9.4 x 10^-8^ | 0.041 (0.007) | 2.2 x 10^-8^ | 67603 | 0.21 |
| *CYP3A7* | rs45446698 | 7 | 99332948 | G/T | 0.04 | - | - | - | - | - | - | - | - | 0.089 (0.016) | 2.4 x 10^-8^ | - | - | - | - |

Effects were aligned to the birth weight-raising allele and are presented in SD units (1 SD of birth weight = 484g (3)). Chr, chromosome; bp, base pair; b37, build 37; EAF, effect allele frequency; Het, heterogeneity; SE, standard error.

* The total number of individuals in the overall meta-analysis for this SNP is lower than the sum of EGG discovery + EGG replication + UK Biobank studies because we excluded discovery studies in which the SNP was imputed with r2_hat or proper_info <0.8.

† Index SNPs available in UK Biobank only, so these are the results for proxy SNPs (r^2^ = 1), available in both UK Biobank and EGG (imputed to HapMap Phase 2) were as follows. No proxy with r^2^ > 0.5 available at *CYP3A7* in HapMap Phase 2.

‡ For the *GCK* locus, SNP rs4607517 (r2=0.73 with rs2971669) was initially followed for replication in EGG due to published fasting glucose association (4) but rs2971669 remained most strongly associated with offspring birth weight in the full meta-analysis (including UK Biobank) and is thus reported here as the index SNP.

**Table S5.** Associations between SNPs at *KCNAB1* and *EBF1* and gestational duration or preterm birth in publicly available data from a GWAS of 43,568 women (1).

| **Locus** | **SNP** | **Alleles (Effect/Other)** | **Maternal SNP association with offspring gestational duration** | | | **Maternal SNP association with preterm birth** | | |
| --- | --- | --- | --- | --- | --- | --- | --- | --- |
|  |  |  |  |  |  |  |  |  |
|  |  |  | **Effect** | **SE** | ***P-*value** | **Effect** | **SE** | ***P-*value** |
| *KCNAB1* | rs7629460 | C/A | 0.48 | 0.12 | 1.1 x 10^-4^ | 0.91 | 0.026 | 4.0 x 10^-4^ |
| *KCNAB1* | rs9872556 | T/C | 0.46 | 0.12 | 1.3 x 10^-4^ | 0.91 | 0.026 | 5.8 x 10^-4^ |
| *EBF1* | rs12520982 | T/C | 1.29 | 0.13 | 2.5 x 10^-21^ | 0.81 | 0.028 | 5.5 x 10^-13^ |
| *EBF1* | rs2964484 | A/G | 1.27 | 0.13 | 5.1 x 10^-21^ | 0.81 | 0.028 | 4.2 x 10^-13^ |

Effects (beta values) were aligned to the birth weight-raising allele from the main meta-analysis and are presented as days (gestational duration) or odds ratio (preterm birth). SE, standard error. *P* values were adjusted for inflation (gestational duration lambda =1.038; preterm birth lambda=1.025).

**Table S6.** Candidate genes at newly identified loci

| **Locus** | **Lead SNP** | **Chr** | **Position (b37)** | **Candidate gene(s) at the locus*** | **Reports on eQTL**** | **Genes +-300kb** | **Literature search for nearby genes of relevance (300kb)** |
| --- | --- | --- | --- | --- | --- | --- | --- |
| *SH2B3* | rs3184504 | 12 | 111884608 | *SH2B3[C,N,E],*  *ATXN2[E],*  *ALDH2[E]* | *ALDH2:* Skin - Sun Exposed (Lower leg) eQTL, *ATXN2:* Whole Blood eQTL, *SH2B3:* Whole Blood eQTL | *CUX2, FAM109A, SH2B3, ATXN2, BRAP, ACAD10* | - |
| *KCNAB1* | rs7629460 | 3 | 155829938 | *KCNAB1[E,N]* | *KCNAB1:* Artery - Aorta eQTL | *SLC33A1, KCNAB1, GMPS, AK130481* | - |
| *TCF7L2* | rs7903146 | 10 | 114758349 | *TCF7L2[N]* | - | *VTI1A, TP11-57H14, TCF7L2* | Hyperglycemia and adverse pregnancy outcome study: common genetic variants in *GCK* and *TCF7L2* are associated with fasting and postchallenge glucose levels in pregnancy and with the new consensus definition of gestational diabetes mellitus from the International Association of Diabetes and Pregnancy Study Groups; PMID 20682688. Bayesian refinement of association signals for 14 loci in 3 common diseases; PMID 23104008. |
| *CYP3A7* | rs45446698 | 7 | 99332948 | *CYP3A7[E,B],*  *ZSCAN25[N,E],* | *CYP3A7:* Adrenal Gland eQTL, *ZKSCAN5:* Skin - Not Sun Exposed (Suprapubic) eQTL | *PTCD1, CPSF4, ATP5J2, AC073063, ZNF789, ZKSCAN5, GS1-259H13, FAM200A, ZSCAN25, CYP3A5, CYP3A7, CYP3A4, CYP3A43, OR2AE1, TRIM4, SNORA40, AZGP1, ZKSCAN1* | Cytochrome P450 allele CYP3A7*1C associated with adverse outcomes in chronic lymphocytic leukemia, breast, and lung cancer; PMID 26964624. Molecular mechanisms of polymorphic *CYP3A7* expression in adult human liver and intestine; PMID 11940601. |

*B=biological candidate, C=coding variant, E=eQTL (from GTEx), N=nearest

** eQTL reported from GTEx v4, GEUVADIS, and 11 other studies for the lead variant using Haploreg v4.1 (http://archive.broadinstitute.org/mammals/haploreg/haploreg.php). When there was no eQTL report for the lead variant, eQTL reports are for the proxy SNPs (r2 >0.8)

**Table S7.** Analysis of index SNPs from maternal GWAS of birth weight conditional on index SNPs from fetal GWAS of birth weight (2) at the at *L3MBTL3* and *GCK* loci.

| **Locus** | **Lead SNP** | **Chr** | **Position (bp, b37)** | **Alleles** | **EAF** | **UK Biobank unconditional** | | | **Conditioned on** | **UK Biobank conditional** | | |
| --- | --- | --- | --- | --- | --- | --- | --- | --- | --- | --- | --- | --- |
|  |  |  |  | **Effect/Other** |  | **Effect (SE)** | ***P-*value** | **N** |  | **Effect (SE)** | ***P-*value** | **N** |
| *L3MBTL3* | rs9375694 | 6 | 130356608 | G/A | 0.31 | 0.033 (0.007) | 1.4 x 10^-6^ | 48632 | rs1415701 | 0.024 (0.008) | 1.2 x 10^-3^ | 48632 |
| *GCK* | rs2971669 | 7 | 44231778 | T/C | 0.23 | 0.032 (0.008) | 3.2 x 10^-5^ | 48632 | rs138715366 | 0.031 (0.008) | 6.8 x 10^-5^ | 48632 |

Effects were aligned to the birth weight-raising allele and are presented in SD units (1 SD of birth weight = 484g (3)). Chr, chromosome; bp, base pair; b37, build 37; EAF, effect allele frequency; SE, standard error.

NB Approximate conditional analysis of the full meta-analysis summary statistics using GCTA (5) produced similar results: *L3MBTL3*: unconditional *P*=2.1 x 10^-9^, conditional P=3.3 x 10^-5^ (N=68,223); *GCK*: unconditional P=5.5 x 10^-9^ conditional P=1.0 x 10^-8^ (N=68,162).

**Table S8.** Associations between maternal genotype and birth weight, adjusted for fetal genoype, and vice versa, in two different study samples: (1) unrelated individuals from the UK Biobank (n=45,436 women with birth weight of first child; n=57,711 participants with own birth weight, using either a linear model (unadjusted for maternal or fetal effects) or a structural equation model (adjusted for both fetal and maternal effects); (2) up to 8705 mother-child pairs from 4 studies: ALSPAC, EFSOCH, DNBC-PTBCTRLS and HAPO (non-GWAS) using linear regression.

| **Locus (lead SNP)** | **Alleles (Effect / Other)** | **Study sample** | **Unadjusted maternal effect** | | **Adjusted maternal effect** | | **Unadjusted fetal effect** | | **Adjusted fetal effect** | |
| --- | --- | --- | --- | --- | --- | --- | --- | --- | --- | --- |
|  |  |  | **Beta (SE)** | **P value** | **Beta (SE)** | **P value** | **Beta (SE)** | **P value** | **Beta (SE)** | **P value** |
| *MTNR1B* (rs10830963) | G/C | UKB unrelated | 0.045 (0.007) | 1.2 x 10^-9^ | 0.046 (0.010) | 7.0 x 10^-6^ | 0.022 (0.007) | 0.001 | -0.002 (0.010) | 0.86 |
|  |  | m/ch pairs (n=8705) | 0.044 (0.015) | 0.004 | 0.036 (0.020) | 0.08 | 0.038 (0.015) | 0.01 | 0.017 (0.020) | 0.4 |
|  |  | **M/A** | **0.045 (0.006)** | **1.6 x10^-12^** | **0.044 (0.009)** | **8.7 x 10^-7^** | **0.025 (0.006)** | **8.9 x 10^-5^** | **0.002 (0.009)** | **0.84** |
| *HMGA2* (rs1351394) | T/C | UKB unrelated | 0.036 (0.007) | 6.9 x 10^-8^ | 0.021 (0.009) | 0.03 | 0.042 (0.006) | 6.8 x 10^-13^ | 0.032 (0.009) | 2.0 x 10^-4^ |
|  |  | m/ch pairs (n=6316) | 0.018 (0.018) | 0.31 | 0.004 (0.018) | 0.82 | 0.031 (0.018) | 0.08 | 0.032 (0.018) | 0.08 |
|  |  | **M/A** | **0.034 (0.007)** | **2.5 x 10^-7^** | **0.018 (0.008)** | **0.03** | **0.041 (0.006)** | **6.7 x 10^-13^** | **0.032 (0.008)** | **7.0 x 10^-5^** |
| *SH2B3* (rs3184504) | C/T | UKB unrelated | 0.032 (0.007) | 1.8 x 10^-6^ | 0.024 (0.009) | 0.01 | 0.025 (0.006) | 1.8 x 10^-5^ | 0.013 (0.009) | 0.12 |
|  |  | m/ch pairs (n=6316) | 0.047 (0.018) | 0.008 | 0.043 (0.018) | 0.02 | -0.017 (0.017) | 0.32 | -0.012 (0.018) | 0.52 |
|  |  | **M/A** | **0.034 (0.007)** | **1.9 x 10^-7^** | **0.028 (0.008)** | **5.5 x 10^-4^** | **0.020 (0.006)** | **3.2 x 10^-4^** | **0.008 (0.008)** | **0.32** |
| *KCNAB1* (rs7629460) | C/A | UKB unrelated | 0.042 (0.007) | 5.4 x 10^-10^ | 0.055 (0.009) | 4.3 x 10^-9^ | -0.0003 (0.006) | 0.96 | -0.028 (0.009) | 0.001 |
|  |  | m/ch pairs (n=6159) | 0.031 (0.018) | 0.09 | 0.026 (0.019) | 0.16 | 0.02 (0.019) | 0.27 | 0.008 (0.019) | 0.66 |
|  |  | **M/A** | **0.041 (0.007)** | **5.1 x10^-10^** | **0.05 (0.008)** | **1.0 x 10^-9^** | **0.002 (0.006)** | **0.79** | **-0.021 (0.008)** | **0.009** |
| *L3MBTL3* (rs9375694) | G/A | UKB unrelated | 0.032 (0.007) | 7.1 x 10^-6^ | 0.026 (0.010) | 0.009 | 0.024 (0.006) | 2.0 x 10^-4^ | 0.01 (0.009) | 0.27 |
|  |  | m/ch pairs (n=6159) | 0.042 (0.019) | 0.02 | 0.022 (0.019) | 0.25 | 0.051 (0.019) | 0.007 | 0.044 (0.019) | 0.02 |
|  |  | **M/A** | **0.033 (0.007)** | **4.3 x 10^-7^** | **0.025 (0.009)** | **0.005** | **0.026 (0.006)** | **3.8 x 10^-6^** | **0.016 (0.008)** | **0.05** |
| *GCK* (rs2971669) | T/C | UKB unrelated | 0.032 (0.008) | 6.4 x 10^-5^ | 0.036 (0.011) | 0.001 | 0.009 (0.007) | 0.21 | -0.009 (0.010) | 0.36 |
|  |  | m/ch pairs (n=8551) | 0.063 (0.016) | 1.0 x 10^-4^ | 0.061 (0.022) | 0.006 | 0.033 (0.017) | 0.05 | 0.003 (0.023) | 0.9 |
|  |  | **M/A** | **0.038 (0.007)** | **9.4 x 10^-8^** | **0.041 (0.01)** | **3.1 x 10^-5^** | **0.012 (0.006)** | **0.05** | **-0.007 (0.009)** | **0.44** |
| *EBF1* (rs12520982) | T/C | UKB unrelated | 0.039 (0.008) | 1.9 x 10^-7^ | 0.033 (0.010) | 0.001 | 0.028 (0.007) | 3.7 x 10^-5^ | 0.011 (0.010) | 0.24 |
|  |  | m/ch pairs (n=6159) | 0.015(0.019) | 0.42 | 0.021 (0.019) | 0.27 | 0.002 (0.019) | 0.91 | -0.012 (0.019) | 0.54 |
|  |  | **M/A** | **0.035 (0.007)** | **1.6 x 10^-6^** | **0.03 (0.009)** | **5.9 x 10^-4^** | **0.025 (0.007)** | **1.5 x 10^-4^** | **0.006 (0.009)** | **0.5** |
| *TCF7L2* (rs7903146) | T/C | UKB unrelated | 0.034 (0.007) | 3.9 x 10^-6^ | 0.031 (0.010) | 0.003 | 0.02 (0.006) | 0.002 | 0.005 (0.009) | 0.57 |
|  |  | m/ch pairs (n=8696) | 0.047 (0.015) | 0.002 | 0.025 (0.022) | 0.26 | 0.045 (0.015) | 0.003 | 0.027 (0.016) | 0.09 |
|  |  | **M/A** | **0.036 (0.006)** | **1.03x10^-8^** | **0.03 (0.009)** | **9.9 x 10^-4^** | **0.023 (0.006)** | **2.6 x 10^-5^** | **0.01 (0.008)** | **0.19** |
| *ACTL9* (rs2918299) | C/T | UKB unrelated | 0.047 (0.009) | 4.9 x 10^-7^ | 0.046 (0.013) | 5.0 x 10^-4^ | 0.027 (0.008) | 0.001 | 0.003 (0.012) | 0.78 |
|  |  | m/ch pairs (n=6159) | 0.082 (0.039) | 0.04 | 0.048 (0.070) | 0.49 | 0.061 (0.019) | 0.001 | 0.046 (0.028) | 0.1 |
|  |  | **M/A** | **0.049 (0.009)** | **2.68x10^-8^** | **0.046 (0.013)** | **3.1 x 10^-4^** | **0.032 (0.007)** | **1.3 x 10^-5^** | **0.010 (0.011)** | **0.38** |
| *CYP3A7* (rs45446698) | G/T | UKB unrelated | 0.088 (0.017) | 1.2 x 10^-7^ | 0.103 (0.023) | 8.1 x 10^-6^ | 0.018 (0.015) | 0.22 | -0.033 (0.021) | 0.13 |
|  |  | m/ch pairs (n=6159) | 0.116 (0.046) | 0.01 | 0.162 (0.055) | 0.003 | 0.005 (0.046) | 0.91 | -0.084 (0.055) | 0.13 |
|  |  | **M/A** | **0.091 (0.016)** | **1.01x10^-8^** | **0.112 (0.021)** | **1.4 x 10^-7^** | **0.017 (0.014)** | **0.24** | **-0.039 (0.020)** | **0.04** |

Effects (beta values) were aligned to the birth weight-raising allele and are presented in SD units (1 SD of birth weight = 484g (3)). SE, standard error; SEM, structural equation model; M/A, meta-analysis (fixed effects, inverse variance). There was little detectable heterogeneity in the meta-analysis (all *P >* 0.05, Bonferroni corrected for 40 tests; median *I^2^ =* 0.0% [inter-quartile range 0.0% to 39.8%]).

**Table S9.** Look-up of fetal genotype associations with birth weight (unadjusted for maternal genotype) in up to 143,675 individuals, for the 10 loci identified in the maternal GWAS of birth weight (unadjusted for fetal genotype).

| **Locus** | **Lead SNP** | **Chr** | **Position (bp, b37)** | **Alleles** | **Maternal GWAS (current study)** | | | | **Fetal GWAS*** | | | | **Difference (Maternal beta - Fetal beta)** |
| --- | --- | --- | --- | --- | --- | --- | --- | --- | --- | --- | --- | --- | --- |
|  |  |  |  | **Effect/Other** | **Beta** | **SE** | ***P*-value** | **N** | **Beta** | **SE** | **P-value** | **N** |  |
|  |  |  |  |  |  |  |  |  |  |  |  |  |  |
| *MTNR1B* | rs10830963 | 11 | 92708710 | G/C | 0.052 | 0.005 | 1.0 x 10^-19^ | 71341 | 0.023 | 0.004 | 2.9 x 10^-8^ | 143663 | 0.029 |
| *HMGA2* | rs1351394 | 12 | 66351826 | T/C | 0.034 | 0.005 | 1.4 x 10^-10^ | 68247 | 0.044 | 0.004 | 1.9 x 10^-32^ | 143671 | -0.01 |
| *SH2B3* | rs3184504 | 12 | 111884608 | C/T | 0.033 | 0.005 | 6.9 x 10^-10^ | 68249 | 0.017 | 0.004 | 2.9 x 10^-6^ | 142404 | 0.016 |
| *KCNAB1* | rs7629460 | 3 | 155829938 | C/A | 0.039 | 0.007 | 1.6 x 10^-9^ | 48632 | 0.009 | 0.004 | 1.7 x 10^-2^ | 139423 | 0.03 |
| *L3MBTL3* | rs9375694 | 6 | 130356608 | G/A | 0.035 | 0.006 | 2.1 x 10^-9^ | 68223 | 0.019 | 0.004 | 1.7 x 10^-6^ | 139427 | 0.016 |
| *GCK* | rs2971669 | 7 | 44231778 | T/C | 0.038 | 0.007 | 5.5 x 10^-9^ | 68162 | 0.010 | 0.005 | 2.8 x 10^-2^ | 143671 | 0.028 |
| *EBF1* | rs12520982 | 5 | 157894747 | T/C | 0.041 | 0.007 | 9.9 x 10^-9^ | 48632 | 0.021 | 0.004 | 3.1 x 10^-7^ | 143669 | 0.02 |
| *TCF7L2* | rs7903146 | 10 | 114758349 | T/C | 0.034 | 0.006 | 1.2 x 10^-8^ | 68253 | 0.007 | 0.004 | 7.7 x 10^-2^ | 143675 | 0.027 |
| *ACTL9* | rs2918299 | 19 | 8787273 | C/T | 0.041 | 0.007 | 2.2 x 10^-8^ | 67603 | 0.028 | 0.005 | 1.4 x 10^-7^ | 131197 | 0.013 |
| *CYP3A7* | rs45446698 | 7 | 99332948 | G/T | 0.089 | 0.016 | 2.4 x 10^-8^ | 48632 | 0.021 | 0.010 | 3.7 x 10^-2^ | 131089 | 0.068 |

Effects (beta values) were aligned to the birth weight-raising allele and are presented in SD units (1 SD of birth weight = 484g (3)). Chr, chromosome; bp, base pair; b37, build 37; SE, standard error.

*These results are look-ups in published summary data from the most recent fetal GWAS of birth weight (European-only meta-analysis (2)).

**Table S11.** Results of gene set enrichment analysis

| **Database** | **Gene set** | **Number of genes (mapped to MAGENTA)** | **95th percentile enrichment cutoff** | | | **75th percentile enrichment cutoff** | | |
| --- | --- | --- | --- | --- | --- | --- | --- | --- |
|  |  |  | **P** | **FDR** | **Expected (observed) number of genes** | **P** | **FDR** | **Expected (observed) number of genes** |
| REACTOME | Xenobiotics | 15(11) | 0.000002 | 0.0003 | 1(6) | 0.03 | 1.0 | 3(6) |
| GOTERM | Drug metabolic process | 15(14) | 0.00003 | 0.01 | 1(6) | 0.002 | 1.0 | 4(9) |
| KEGG | Linoleic acid metabolism | 29(21) | 0.0004 | 0.02 | 1(6) | 0.02 | 0.25 | 5(10) |
| Ingenuity | PXR/RXR activation | 49(45) | 0.0002 | 0.03 | 2(9) | 0.008 | 0.25 | 11(19) |
| Ingenuity | GM-CSF signaling | 23(20) | 0.002 | 0.04 | 1(5) | 0.22 | 0.55 | 5(7) |
| Ingenuity | Neurotrophin. TRK signalling | 16(15) | 0.004 | 0.04 | 1(4) | 0.31 | 0.64 | 4(5) |
| PANTHER Molecular function | Other actin family cytoskeletal protein | 31(22) | 0.0003 | 0.04 | 1(6) | 0.003 | 0.11 | 6(12) |

Analysis of the overall GWAS summary data to identify enrichment in biological pathways. Top results (FDR < 0.05 at the 95th percentile enrichment threshold) from a total of 3230 pathways tested for enrichment of multiple modest associations between maternal genotype and offspring birth weight.

**Table S12.** Details of proxy SNPs analysed in EGG follow-up studies if the index SNP was unavailable

| **LEAD SNP** | **NEAREST GENE** | **BEST PROXY SNP** | **R2 (1000G PILOT1 CEU)** | **2ND-BEST PROXY SNP** | **R2 (1000G PILOT1 CEU)** |
| --- | --- | --- | --- | --- | --- |
| rs1920773 | *KERA* | rs1861129 | 1 | rs4842749 | 1 |
| rs17065363 | *LOC285501* | rs62342764 | 1 | rs17065376 | 1 |
| rs11811998 | *MYCL1* | rs72939665 | 1 | rs34387655 | 1 |
| rs10830963 | *MTNR1B* | rs11020124 | NA | NA |  |
| rs4849902 | *INHBB* | NA |  | NA |  |
| rs6692555 | *PLEKHM2* | rs72878883 | 1 | rs6674904 | 1 |
| rs7721459 | *GRAMD3* | rs6879479 | 1 | rs28580210 | 0.908 |
| rs311565 | *POLR1A* | rs311566 | 1 | rs311567 | 1 |
| rs989264 | *NEDD1* | rs989265 | 1 | rs76003827 | 1 |
| rs838324 | *TBX3* | NA |  | NA |  |
| rs12056975 | *ANKS6* | rs10987747 | 1 | rs33998698 | 1 |
| rs9549119 | *FOXO1* | NA |  | NA |  |
| rs3887673 | *CMTM7* | rs77036319 | 1 | rs2304594 | 1 |
| rs17384756 | *EPB41L3* | rs4798380 | 1 | rs4798381 | 1 |
| rs2748343 | *GRAMD4* | rs2748341 | 0.905 | rs2748338 | 0.87 |
| rs4607517 | *GCK* | rs1799884 | 1 | rs2908286 | 1 |
| rs204928 | *LMO1* | rs204926 | 0.967 | rs110420 | 0.935 |
| rs7972086 | *RAD51AP1* | rs7314915 | 1 | rs2907492 | 1 |

**References**

1 Zhang, G., Feenstra, B., Bacelis, J., Liu, X., Muglia, L.M., Juodakis, J., Miller, D.E., Litterman, N., Jiang, P.P., Russell, L. *et al.* (2017) Genetic Associations with Gestational Duration and Spontaneous Preterm Birth. *N Engl J Med*.

2 Horikoshi, M., Beaumont, R.N., Day, F.R., Warrington, N.M., Kooijman, M.N., Fernandez-Tajes, J., Feenstra, B., van Zuydam, N.R., Gaulton, K.J., Grarup, N. *et al.* (2016) Genome-wide associations for birth weight and correlations with adult disease. *Nature*, **538**, 248-252.

3 Horikoshi, M., Yaghootkar, H., Mook-Kanamori, D.O., Sovio, U., Taal, H.R., Hennig, B.J., Bradfield, J.P., St Pourcain, B., Evans, D.M., Charoen, P. *et al.* (2013) New loci associated with birth weight identify genetic links between intrauterine growth and adult height and metabolism. *Nat. Genet.*, **45**, 76-82.

4 Dupuis, J., Langenberg, C., Prokopenko, I., Saxena, R., Soranzo, N., Jackson, A.U., Wheeler, E., Glazer, N.L., Bouatia-Naji, N., Gloyn, A.L. *et al.* (2010) New genetic loci implicated in fasting glucose homeostasis and their impact on type 2 diabetes risk. *Nat. Genet.*, **42**, 105-116.

5 Yang, J., Ferreira, T., Morris, A.P., Medland, S.E., Genetic Investigation of, A.T.C., Replication, D.I.G., Meta-analysis, C., Madden, P.A., Heath, A.C., Martin, N.G. *et al.* (2012) Conditional and joint multiple-SNP analysis of GWAS summary statistics identifies additional variants influencing complex traits. *Nat Genet*, **44**, 369-375, S361-363.

**Additional acknowledgements by study:**

**ALSPAC:** We are extremely grateful to all of the families who took part in this study, the midwives for their help in recruiting them, and the whole ALSPAC team, which includes interviewers, computer and laboratory technicians, clerical workers, research scientists, volunteers, receptionists, managers and nurses.

**1958BC-WTCCC and 1958BC-T1DGC.** This work made use of data and samples generated by the 1958 Birth Cohort (NCDS) , which is managed by the Centre for Longitudinal Studies at the UCL Institute of Education, The authors are deeply grateful to the 1958 birth cohort participants for their longstanding commitment and support, and to all staff for cohort coordination and data collection.

**CHOP:** The authors thank the network of primary care clinicians and the patients and families for their contribution to this project and to clinical research facilitated by the Pediatric Research Consortium (PeRC) at The Children’s Hospital of Philadelphia. R. Chiavacci, E. Dabaghyan, A. (Hope) Thomas, K. Harden, A. Hill, C. Johnson-Honesty, C. Drummond, S. Harrison, F. Salley, C. Gibbons, K. Lilliston, C. Kim, E. Frackelton, F. Mentch, G. Otieno, K. Thomas, C. Hou, K. Thomas and M.L. Garris provided expert assistance with genotyping and/or data collection and management. The authors would also like to thank S. Kristinsson, L.A. Hermannsson and A. Krisbjörnsson of Raförninn ehf for extensive software design and contributions.

**COPSAC:** We gratefully express our gratitude to the children and families of the COPSAC2000 cohort study for all their support and commitment. We acknowledge and appreciate the unique efforts of the COPSAC research team.

**DNBC-PTB-CTRL:** We are very grateful to all DNBC families who took part in the study. We would also like to thank everyone involved in data collection and biological material handling.

**Gen3G:** Gen3G investigators would like to acknowledge the participants, the research staff from the endocrinology group at the Centre de Recherche clinique of the Centre Hospitalier de l’Université de Sherbrooke (CR-CHUS), the clinical and research staff of the Clinique de Prélèvement en Grossesse du CHUS, and the clinical staff from the CHUS Obstetric Department. The CR-CHUS is a FRQ-Sante affiliated research centre; recruitment and follow-up of Gen3G participants was possible based on the on-going support from the CR-CHUS.

**Generation R:** The Generation R Study is conducted by the Erasmus Medical Center in close collaboration with the School of Law and Faculty of Social Sciences of the Erasmus University Rotterdam, the Municipal Health Service Rotterdam area, Rotterdam, the Rotterdam Homecare Foundation, Rotterdam and the Stichting Trombosedienst and Artsenlaboratorium Rijnmond (STAR-MDC), Rotterdam. We gratefully acknowledge the contribution of participating mothers, general practitioners, hospitals, midwives and pharmacies in Rotterdam. The generation and management of GWAS genotype data for the Generation R Study were done at the Genetic Laboratory of the Department of Internal Medicine, Erasmus MC, The Netherlands. We would like to thank Karol Estrada, Dr. Tobias A. Knoch, Anis Abuseiris, Luc V. de Zeeuw, and Rob de Graaf, for their help in creating GRIMP, BigGRID, MediGRID, and Services@MediGRID/D-Grid, (funded by the German Bundesministerium fuer Forschung und Technology; grants 01 AK 803 A-H, 01 IG 07015 G) for access to their grid computing resources. We thank Mila Jhamai, Manoushka Ganesh, Pascal Arp, Marijn Verkerk, Lizbeth Herrera and Marjolein Peters for their help in creating, managing and QC of the GWAS database. Also, we thank Karol Estrada for his support in creation and analysis of imputed data.

**HAPO:** HAPO would like to acknowledge the participants and research personnel at the participating HAPO field centres.

**MoBa:** We are grateful to all the participating families in Norway who take part in this ongoing cohort study. Researchers interested in using MoBa data must obtain approval from the Scientific Management Committee of MoBa and from the Regional Committee for Medical and Health Research Ethics for access to data and biological material. Researchers are required to follow the terms of a Data or Material Transfer Agreement containing a number of clauses designed to ensure protection of privacy and compliance with relevant laws.

**NCCGP:** We thank all of the participants of the North Cumbria Community Genetics Project and previous members of the study team, in particular Professor Sir John Burn and Mrs Pat Jonas.

**NTR:** The Netherlands Twin Register warmly thanks all participating twin families for their contributions.

**QIMR:** We thank Richard Parker, Soad Hancock, Judith Moir, Sally Rodda, Pieta-Maree Shertock, Heather Park, Jill Wood, Pam Barton, Fran Husband, Adele Somerville, Ann Eldridge, Marlene Grace, Kerrie McAloney, Anjali Henders, Lisa Bowdler, Alexandre Todorov, Steven Crooks, David Smyth, Harry Beeby, and Daniel Park. Finally, we thank the twins and their families for their participation.

**Raine:** The authors are grateful to the Raine Study participants and their families, and to the Raine Study research staff for cohort coordination and data collection. The authors gratefully acknowledge the NH&MRC for their long term contribution to funding the study over the last 25 years and also the following Institutions for providing funding for Core Management of the Raine Study: The University of Western Australia (UWA), Raine Medical Research Foundation, UWA Faculty of Medicine, Dentistry and Health Sciences, The Telethon Kids Institute, Women and Infants Research Foundation, Curtin University and Edith Cowan University. The authors gratefully acknowledge the assistance of the Western Australian DNA Bank (National Health and Medical Research Council of Australia National Enabling Facility). This work was supported by resources provided by the Pawsey Supercomputing Centre with funding from the Australian Government and the Government of Western Australia.

**SWS:** We thank the mothers of the Southampton Women's Survey who gave us their time and acknowledge the work of the Southampton Women's Survey Study Group, and the team of dedicated research nurses and ancillary staff for their assistance.

**Details of funding by study**

**ALSPAC:** The work undertaken in this paper was funded by the ERC (Grant number 669545; DevelopObese), US National Institute of Diabetes and Digestive and Kidney Diseases (R01 DK10324) and the Wellcome Trust (WT088806), with the WT088806 grant also providing funds for completion of genome wide genotyping on the ALSPAC mothers. ALSPAC offspring GWAS data was generated by Sample Logistics and Genotyping Facilities at the Wellcome Trust Sanger Institute and LabCorp (Laboratory Corportation of America) using support from 23andMe. Additional maternal data were funded by the British Heart Foundation (SP/07/008/24066) and WellcomeTrust (WT087997). The UK Medical Research Council and Wellcome Trust (Grant ref: 102215/2/13/2) and the University of Bristol provide core support for ALSPAC. This publication is the work of the authors and R. M. Freathy and D. A. Lawlor will serve as guarantors for the contents of this paper.

**BBC:** The Berlin Birth Cohort study was funded by the Deutsche Forschungsgemeinschaft (DFG), Else Kröner-Fresenius Foundation, Jackstädt-Foundation and a research grant of the University of Potsdam, Germany. Details of the study are provided in: Pharmacogenet Genomics. 2009;19(9):710-8.; Circulation. 2006 Oct 17;114(16):1687-92 and Lancet. 2000 Apr 8;355(9211):1241-2. We deeply acknowledge the contribution of the participating families. Genotyping and analyses were supported by Wellcome Trust (grant numbers WT064890, WT090532, WT098381) and Framework VII: ENGAGE (HEALTH-F4-2007-201413).

**1958BC-WTCCC:**Statistical analyses were funded by the Academy of Finland (Project 24300796 and SALVE/PREVMEDSYN). The management of the 1958 Birth Cohort is funded by the Economic and Social Research Council (grant number ES/M001660/1). Access to these resources was enabled via the 58READIE Project funded by Wellcome Trust and Medical Research Council (grant numbers WT095219MA and G1001799). DNA collection was funded by MRC grant G0000934 and cell-line creation by Wellcome Trust grant 068545/Z/02. This study makes use of data generated by the Wellcome Trust Case-Control Consortium. A full list of investigators who contributed to generation of the data is available from the Wellcome Trust Case-Control Consortium website. Funding for the project was provided by the Wellcome Trust under the award 076113. Great Ormond Street Hospital/University College London, Institute of Child Health receives a proportion of funding from the Department of Health's National Institute for Health Research (NIHR) ('Biomedical Research Centres' funding).

**1958BC-T1DGC:**Statistical analyses were funded by the Academy of Finland (Project 24300796 and SALVE/PREVMEDSYN). The management of the 1958 Birth Cohort is funded by the Economic and Social Research Council (grant number ES/M001660/1). Access to these resources was enabled via the 58READIE Project funded by Wellcome Trust and Medical Research Council (grant numbers WT095219MA and G1001799).  DNA collection was funded by MRC grant G0000934 and cell-line creation by Wellcome Trust grant 068545/Z/02. This research used resources provided by the Type 1 Diabetes Genetics Consortium, a collaborative clinical study sponsored by the National Institute of Diabetes and Digestive and Kidney Diseases (NIDDK), National Institute of Allergy and Infectious Diseases, National Human Genome Research Institute, National Institute of Child Health and Human Development, and Juvenile Diabetes Research Foundation International (JDRF) and supported by U01 DK062418. Great Ormond Street Hospital/University College London, Institute of Child Health receives a proportion of funding from the Department of Health's National Institute for Health Research (NIHR) ('Biomedical Research Centres' funding).

**CHOP:** This research was financially supported by an Institute Development Award from the Children’s Hospital of Philadelphia, a Research Development Award from the Cotswold Foundation and NIH grant R01 HD056465.

**COPSAC2000:** COPSAC is funded by private and public research funds listed on www.copsac.com. The Lundbeck Foundation; The Danish Strategic Research Council; the Pharmacy Foundation of 1991; the Danish Medical Research Council and The Danish Pediatric Asthma Centre provided the core support for COPSAC research center.

**DNBC-GOYA:** The genotyping for GOYA was funded by the Wellcome Trust (WT 084762). GOYA is a nested study within The Danish National Birth Cohort which was established with major funding from the Danish National Research Foundation. Additional support for this cohort has been obtained from the Pharmacy Foundation, the Egmont Foundation, The March of Dimes Birth Defects Foundation, the Augustinus Foundation, and the Health Foundation.

**DNBC-PTB-CTRL:** The Danish National Birth Cohort (DNBC) is a result of major grants from the Danish National Research Foundation, the Danish Pharmacists’ Fund, the Egmont Foundation, the March of Dimes Birth Defects Foundation, the Augustinus Foundation, and the Health Fund of the Danish Health Insurance Societies. The DNBC biobank is a part of the Danish National Biobank resource, which is supported by the Novo Nordisk Foundation. The generation of GWAS genotype data for the DNBC samples was carried out within the Gene Environment Association Studies (GENEVA) consortium with funding provided through the National Institutes of Health’s Genes, Environment, and Health Initiative (U01HG004423; U01HG004446; U01HG004438).

**EFSOCH:** The Exeter Family Study of Childhood Health (EFSOCH) was supported by South West NHS Research and Development, Exeter NHS Research and Development, the Darlington Trust and the Peninsula National Institute of Health Research (NIHR) Clinical Research Facility at the University of Exeter. The views expressed are those of the authors and not necessarily those of NIHR, the NHS or the Department of Health.

**FHS:** The Framingham Heart Study phenotype-genotype analyses were supported by the National Institute of Aging, R01AG29451. This research was conducted using data and resources from the Framingham Heart Study of the National Heart Lung and Blood Institute of the National Institutes of Health and Boston University School of Medicine based on analyses by Framingham Heart Study investigators participating in the SNP Health Association Resource (SHARe) project. This work was supported by the National Heart, Lung and Blood Institute's Framingham Heart Study (Contract No. N01-HC-25195) and its contract with Affymetrix, Inc for genotyping services (Contract No.N02-HL-6-4278). A portion of this research utilized the Linux Cluster for Genetic Analysis (LinGA-II) funded by the Robert Dawson Evans Endowment of the Department of Medicine at Boston University School of Medicine and Boston Medical Center.

**Gen-3G:** The Gen3G prospective cohort was supported by the Fonds de Recherche du Québec – Santé (FRQS – subvention Fonctionnement – Recherche Clinique - grant #20697) and by a Canadian Institute of Health Research (CIHR) Operating grant (Institute of Nutrition, Metabolism and Diabetes; MOP- 115071).

**Generation R:** The general design of Generation R Study is made possible by ﬁnancial support from the Erasmus Medical Center, Rotterdam, the Erasmus University Rotterdam, the Netherlands Organization for Health Research and Development (ZonMw), the Netherlands Organisation for Scientific Research (NWO), the Ministry of Health, Welfare and Sport and the Ministry of Youth and Families. This research also received funding from the European Union’s Seventh Framework Programme (FP7/2007–2013), project EarlyNutrition under grant agreement n°289346. VWJ received an additional grant from the Netherlands Organization for Health Research and Development (VIDI 016.136.361). This project also received funding from the European Union’s Horizon 2020 research and innovation programme under grant agreements No 633595 (DynaHEALTH) and No 733206 (LIFECYCLE), and from the European Research Council (ERC Consolidator Grant, ERC-2014-CoG-648916 to V.W.V. J.).

**HAPO:** This study was supported by National Institutes of Health (NIH) grants HD34242, HD34243, HG004415, DK097534 and DK099820

**INMA:** This study was funded by grants from Instituto de Salud Carlos III (CB06/02/0041, FIS PI041436, PI081151, PI041705, PS09/00432, FIS-FEDER 03/1615, 04/1509, 04/1112, 04/1931 , 05/1079, 05/1052, 06/1213, 07/0314, 09/02647, PI06/0867, PI09/0009), Spanish Ministry of Science and Innovation (SAF2013-49108-R), European Commission (ENGAGE project and grant agreement HEALTH-F4-2007-201413), Fundació La Marató de TV3, Generalitat de Catalunya-CIRIT AGAUR 2014 SGR-1138, Conselleria de Sanitat Generalitat Valenciana, Departamento de Sanidad del Gobierno Vasco (110093/2005, 110669/2009), Diputación Foral de Gipuzkoa (DFG 05/007,DFG06/004, DFG08/001), Ayuntamiento de la Zona del estudio (Azpeitia, Azkoitia, Beasain, Zumarraga, Legazpia, Urretxu). Part of the DNA extractions and genotyping was performed at the Spanish National Genotyping Centre (CEGEN-Barcelona).

**MoBa:**This work was supported by grants from the Norwegian Research Council (FUGE 183220/S10, FRIMEDKLI-05 ES236011), Swedish Medical Society (SLS 2008- 21198), Jane and Dan Olsson Foundations and Swedish government grants to researchers in the public health service (ALFGBG-2863, ALFGBG-11522), and the European Community’s Seventh Framework Programme (FP7/2007-2013), ENGAGE Consortium, grant agreement HEALTH-F4-2007-201413. The Norwegian Mother and Child Cohort Study was also supported by the Norwegian Ministry of Health and the Ministry of Education and Research, NIH/NIEHS (contract no N01-ES-75558), NIH/NINDS (grant no.1 UO1 NS 047537-01 and grant no.2 UO1 NS 047537-06A1), and the Norwegian Research Council/FUGE (grant no. 151918/S10). We are grateful to all the participating families in Norway who take part in this on-going cohort study.

**NCCGP:** Funding for the initial setup and maintenance of the NCCGP resource was provided by British Nuclear Fuels plc and orchestrated by the NCCGP management team and staff at Westlakes Research Institute. Subsequent funding for its continued storage and maintenance has been provided from a number of sources, largely project grants utilising samples from the collection.

**NFBC1966:** Academy of Finland (Grant no. 1114194, Grant no. 12926901), University Hospital Oulu and Unit of Primary Care, Biocenter, University of Oulu, and the European Commission (EURO-BLCS, Framework 5 award QLG1-CT-2000-01643 and EU FP7- EurHEALTHAgeing - European Research on Developmental, Birth and Genetic Determinants of Ageing. Grant no. 24000522). Medical Research Council Medical Research Council, UK (G0500539, G0600705, PrevMetSyn/SALVE, PS0476)

**NTR:** Funding was obtained from the Netherlands Organization for Scientific Research (NWO: MagW/ZonMW grants 904-61-090, 985-10-002,904-61-193,480-04-004, 400-05-717, Addiction-31160008 Middelgroot-911-09-032, Spinozapremie 56-464-14192), Center for Medical Systems Biology (CMSB, NWO Genomics), NBIC/BioAssist/RK(2008.024), Biobanking and Biomolecular Resources Research Infrastructure (BBMRI –NL), the VU University’s Institute for Health and Care Research (EMGO+ ) and Neuroscience Campus Amsterdam (NCA), the European Science Foundation (ESF, EU/QLRT-2001-01254), the European Community's Seventh Framework Program (FP7/2007-2013), ENGAGE (HEALTH-F4-2007-201413); the European Research Council (ERC Advanced, 230374), Rutgers University Cell and DNA Repository (NIMH U24 MH068457-06), and the National Institutes of Health (NIH, R01D0042157-01A). Part of the genotyping and analyses were funded by the Genetic Association Information Network (GAIN) of the Foundation for the US National Institutes of Health, and by grants from GAIN and the NIMH (MH081802).

**QIMR:** Supported by National Institutes of Health Grants AA07535, AA0758O, AA07728, AA10249, AA13320, AA13321, AA14041, AA11998, AA17688, DA012854, DA018267, DA018660, DA23668 and DA019951; by Grants from the Australian National Health and Medical Research Council (241944, 339462, 389927, 389875, 389891, 389892, 389938, 442915, 442981, 496739, 552485, 552498, and 628911); by Grants from the Australian Research Council (A7960034, A79906588, A79801419, DP0770096, DP0212016, and DP0343921); and by the 5th Framework Programme (FP-5) GenomEUtwin Project (QLG2-CT-2002-01254).

**RAINE:** This study was supported by the Raine Medical Research Foundation, National Health and Medical Research Council of Australia (NHMRC) and the Canadian Institutes of Health Research (CIHR).

**RHEA:** The Rhea study was financially supported by European projects (EU FP6-2003-Food-3-NewGeneris, EU FP6. STREP Hiwate, EU FP7 ENV.2007.1.2.2.2. Project No 211250 Escape, EU FP7-2008-ENV-1.2.1.4 Envirogenomarkers, EU FP7-HEALTH-2009- single stage CHICOS, EU FP7 ENV.2008.1.2.1.6. Proposal No 226285 ENRIECO, EU- FP7- HEALTH-2012 Proposal No 308333 HELIX), MeDALL (FP7 European Union project, No. 264357), and the Greek Ministry of Health (Program of Prevention of obesity and neurodevelopmental disorders in preschool children, in Heraklion district, Crete, Greece: 2011-2014; “Rhea Plus”: Primary Prevention Program of Environmental Risk Factors for Reproductive Health, and Child Health: 2012-15).

**SWS Mothers:** This work was supported by grants from the Medical Research Council, Dunhill Medical Trust, National Institute for Health Research Southampton Biomedical Research Centre, University of Southampton and University Hospital Southampton National Health Service Foundation Trust, and the European Union’s Seventh Framework Programme (FP7/2007-2013), project EarlyNutrition (grant 289346).

**TwinsUK:** The study was funded by the Wellcome Trust; European Community’s Seventh Framework Programme (FP7/2007-2013). The study also receives support from the National Institute for Health Research (NIHR)- funded BioResource, Clinical Research Facility and Biomedical Research Centre based at Guy's and St Thomas' NHS Foundation Trust in partnership with King's College London. Tim Spector is holder of an ERC Advanced Principal Investigator award*. SNP Genotyping was performed by The Wellcome Trust Sanger Institute and National Eye Institute via NIH/CIDR.

**Early Growth Genetics (EGG) Consortium Members**

Linda S Adair^1^, Tarunveer S Ahluwalia^2,3,4^, Peter Almgren^5^, Wei Ang^6^, Mustafa Atalay^7^, Jonas Bacelis^8^, Robin N Beaumont^9^, Jacques Beckmann^10^, Nienke Bergen^11^, Hans Bisgaard^2^, Thomas Bond^12^, Dorret I Boomsma^13^, Jonathan P Bradfield^14^, Mariona Bustamante^15,16,17,18^, Alana Cavadino^19^, Lachlan Coin^20^, Cyrus Cooper^21^, Diana L Cousminer^22,23,24^, Shikta Das^25^, Felix R Day^26^, George V Dedoussis^27^, Paul Elliott^12^, Johan G Eriksson^28,29,30^, David M Evans^31,32,33^, Bjarke Feenstra^34^, Janine F Felix^11,35,36^, Timothy M Frayling^9^, Rachel M Freathy^9,31^, Romy Gaillard^35^, Frank Geller^34^, Matthew Gillman^37^, Struan FA Grant^14,23,38,39^, Niels Grarup^3^, Maria M Groen-Blokhuis^40^, Leif Groop^5,22^, Monica Guxens^16,17,18^, Dexter Hadley^41^, Hakon Hakonarson^14,23,38^, Torben Hansen^3^, Andrew T Hattersley^9^, Geoff Hayes^42,43^, Joachim Heinrich^44,45^, Øyvind Helgeland^46^, Tine Brink Henrisksen^47^, Anke Hinney^48^, Joel Hirschhorn^49^, Marie-France Hivert^50^, Berthold Hocher^51,52,53^, John WV Holloway^54^, Momoko Horikoshi^55,56^, Jouke-Jan Hottenga^13^, Ville Huikari^57^, Elina Hyppönen^58,59,60^, Bo Jacobsson^61,62^, Vincent WV Jaddoe^11,35,36^, Marjo-Riitta Jarvelin^57,63,64,65^, Stefan Johansson^46^, Marika Kaakinen^66^, Marjan Kerkhof^67^, Antje Körner^68,69^, Sailesh Kotecha^70^, Eskil Kreiner^2^, Zoltán Kutalik^10,71^, Timo A Lakka^7,72,73^, Leslie Lange^74^, Debbie A Lawlor^31,32^, Terho Lehtimäki^75,76^, Alexandra M Lewin^63^, Cecilia M Lindgren^55,77^, Virpi Lindi^7^, Allan Linneberg^78^, William L Lowe Jr^42^, Ronald Ching Wan Ma^79,80,81^, Aurelien Mace^82^, Reedik Mägi^83^, Per Magnus^61^, Anubha Mahajan^55^, Julie A Marsh^6^, Mark I McCarthy^55,56,84^, Niina McCarthy^85^, Mads Melbye^34,86,87^, Karen L Mohlke^88^, Dennis O Mook-Kanamori^89,90,91^, Andrew P Morris^55,83,92^, Jeff Murray^93^, Ronny Myhre^61^, Pål R Njølstad^46^, Ellen Aagaard Nøhr^94^, Ioanna Ntalla^27,95^, Paul O'Reilly^96,97^, Emily Oken^37^, Ken K Ong^26,98^, Kalliope Panoutsopoulou^99^, Oluf Pedersen^3^, Craig E Pennell^6^, John RB Perry^26^, Pimphen Charoen^63,100^, Niina Pitkänen^101^, Beate St Pourcain^31,102^, Christine Power^58^, Rashmi Prasad^5^, Inga Prokopenko^55,66^, Olli T Raitakari^101,103^, Rebecca M Reynolds^104^, Rebecca Richmond^31^, Alina Rodriguez^63,105^, Rany Salem^106,107,108,109^, Seang-Mei Saw^110,111^, Sylvain Sebert^57,64^, Verena Sengpiel^112^, Thorkild IA Sørensen^31,32,113,114^, Ulla Sovio^115^, Evie Stergiakouli^31^, David P Strachan^116^, Jordi Sunyer^16,17,18,117^, H Rob Taal^11^, Yik-Ying Teo^110,118,119^, Elisabeth Thiering^44,120^, Nicholas J Timpson^31,32^, Jessica Tyrrell^9,121^, André G Uitterlinden^11,35,122^, Cornelia M van Duijn^35^, Tanja G M Vrijkotte^123^, Carol A Wang^6^, Nicole M Warrington^6,33^, William J Watkins^70^, Erich Wichmann^44^, Elisabeth E Widén^22^, Gonneke Willemsen^13^, James F Wilson^124,125^, Hanieh Yaghootkar^9^, Mohammad Hadi Zafarmand^123^, Eleftheria Zeggini^99^

1. Department of Nutrition, University of North Carolina, Chapel Hill, NC 27599, USA.

2. COPSAC, Copenhagen Prospective Studies on Asthma in Childhood, Herlev and Gentofte Hospital, University of Copenhagen, Copenhagen, 2900 Hellerup, Denmark.

3. The Novo Nordisk Foundation Center for Basic Metabolic Research, Section of Metabolic Genetics, Faculty of Health and Medical Sciences, University of Copenhagen, Copenhagen, DK-2100, Denmark.

4. Steno Diabetes Center, Gentofte, DK-2820, Denmark.

5. Department of Clinical Sciences, Diabetes and Endocrinology, Lund University Diabetes Centre, Malmö, 205 02, Sweden.

6. School of Women’s and Infants’ Health, The University of Western Australia, Perth, WA 6009, Australia.

7. Institute of Biomedicine, Physiology, University of Eastern Finland, Kuopio, FI-70211, Finland.

8. Department of Obstetrics and Gynecology, Sahlgrenska University Hospital, Gothenburg, 41685, Sweden.

9. Institute of Biomedical and Clinical Science, University of Exeter Medical School, Royal Devon and Exeter Hospital, Exeter, EX2 5DW, UK.

10. Swiss Institute of Bioinformatics, Lausanne, CH-1015, Switzerland.

11. The Generation R Study Group, Erasmus MC, University Medical Center Rotterdam, 3015 CE, the Netherlands.

12. Department of Epidemiology and Biostatistics, School of Public Health, Imperial College London, London, W2 1PG, UK.

13. Netherlands Twin Register, Department of Biological Psychology, VU University, Amsterdam, 1081 BT, the Netherlands.

14. Center for Applied Genomics, The Children’s Hospital of Philadelphia, Philadelphia, PA 19104, USA.

15. Center for Genomic Regulation (CRG), Barcelona, 08003, Spain.

16. ISGlobal, Centre for Research in Environmental Epidemiology (CREAL), Barcelona, 08003, Spain.

17. Universitat Pompeu Fabra (UPF), Barcelona, 08002, Spain.

18. CIBER de Epidemiología y Salud Pública (CIBERESP), 28029, Spain.

19. Centre for Environmental and Preventive Medicine, Wolfson Institute of Preventive Medicine, Queen Mary University of London, London, EC1M 6BQ, UK.

20. Institute for Molecular Bioscience, University of Queensland, Brisbane, QLD 4072, Australia.

21. MRC Lifecourse Epidemiology Unit, Faculty of Medicine, University of Southampton, Southampton, SO16 6YD, UK.

22. Institute for Molecular Medicine, Finland (FIMM), University of Helsinki, Helsinki, FI-00014, Finland.

23. Division of Human Genetics, The Children’s Hospital of Philadelphia, Philadelphia, PA 19104, USA.

24. Department of Genetics, Perelman School of Medicine, University of Pennsylvania, Philadelphia, PA 19104, USA.

25. Department of Primary Care and Public Health, Imperial College London, Hammersmith, W6 6RP, UK.

26. MRC Epidemiology Unit, University of Cambridge School of Clinical Medicine, Cambridge, CB2 0QQ, UK.

27. Department of Nutrition and Dietetics, School of Health Science and Education, Harokopio University, Athens, 17671, Greece.

28. National Institute for Health and Welfare, Helsinki, 00271, Finland.

29. Department of General Practice and Primary Health Care, University of Helsinki and Helsinki University Hospital, Helsinki, 00014, Finland.

30. Folkhälsan Research Center, Helsinki, 00250, Finland.

31. Medical Research Council Integrative Epidemiology Unit at the University of Bristol, Bristol, BS8 2BN, UK.

32. School of Social and Community Medicine, University of Bristol, Bristol, BS8 2BN, UK.

33. The University of Queensland Diamantina Institute, Translational Research Institute, Brisbane, QLD 4102, Australia.

34. Department of Epidemiology Research, Statens Serum Institute, Copenhagen, DK-2300, Denmark.

35. Department of Epidemiology, Erasmus MC, University Medical Center Rotterdam, 3015 CE, the Netherlands.

36. Department of Pediatrics, Erasmus MC, University Medical Center Rotterdam, 3015 CE, the Netherlands.

37. Obesity Prevention Program, Department of Population Medicine, Harvard Medical School and Harvard Pilgrim Health Care Institute, Boston, MA 02215, USA.

38. Department of Pediatrics, Perelman School of Medicine, University of Pennsylvania, Philadelphia, PA 19104, USA.

39. Division of Endocrinology, The Children’s Hospital of Philadelphia, Philadelphia, PA 19104, USA.

40. Department of Biological Psychology, VU University Amsterdam, NCA Neuroscience Campus Amsterdam, EMGO+ Institute for Health and Care Research, Amsterdam, 1081 HV, the Netherlands.

41. Department of Pediatrics, University of California San Francisco School of Medicine, San Francisco, CA 94158, USA.

42. Department of Medicine, Division of Endocrinology, Metabolism, and Molecular Medicine, Feinberg School of Medicine, Northwestern University, Chicago, IL 60611, USA.

43. Center for Genetic Medicine, Northwestern University Feinberg School of Medicine, Chicago, IL 60611, USA.

44. Institute of Epidemiology I, Helmholtz Zentrum München- German Research Center for Environmental Health, Neuherberg, 85764, Germany.

45. Institute and Outpatient Clinic for Occupational, Social and Environmental Medicine, Inner City Clinic, University Hospital Munich, Ludwig Maximilian University of Munich, Munich, Germany.

46. KG Jebsen Center for Diabetes Research, Department of Clinical Science, University of Bergen, Children's Hospital, Bergen, N-5009, Norway.

47. Department of Clinical Medicine - Department of Paediatrics, Aarhus University Hospital, Aarhus N, 8200, Denmark.

48. Department of Child and Adolescent Psychiatry, Psychosomatics and Psychotherapy, University Hospital Essen, University of Duisburg-Essen, Essen, 45147, Germany.

49. Broad Institute of MIT and Harvard, Cambridge, MA 02142, USA.

50. Department of Medicine, Universite de Sherbrooke, Sherbrooke, QC J1K 2R1, Canada.

51. Institute of Nutritional Sciences, University of Potsdam, Potsdam, D-14558, Germany.

52. IFLb GmbH - Institute for Laboratory Medicine, Berlin, D-10627, Germany.

53. Department of Basic Medicine, Medical College of Hunan Normal University, Changsha, 46000, China.

54. Human Development and Health, Faculty of Medicine, University of Southampton, Southampton, SO16 6YD, UK.

55. Wellcome Trust Centre for Human Genetics, University of Oxford, Oxford, OX3 7BN, UK.

56. Oxford Centre for Diabetes, Endocrinology and Metabolism, University of Oxford, Oxford, OX3 7LE, UK.

57. Biocenter Oulu, University of Oulu, 90220, Finland.

58. Population, Policy and Practice, UCL Institute of Child Health, University College London, London, WC1N 1EH, UK.

59. Centre for Population Health Research, School of Health Sciences, and Sansom Institute, University of South Australia, Adelaide, SA 5001, Australia.

60. South Australian Health and Medical Research Institute, Adelaide, SA 5000, Australia.

61. Norwegian Institute of Public Health, Oslo, N-0403, Norway.

62. Department of Obstetrics and Gynecology, Sahlgrenska Academy, University of Gothenburg, Gothenburg, SE 41685, Sweden.

63. Department of Epidemiology and Biostatistics, MRC–PHE Centre for Environment & Health, School of Public Health, Imperial College London, London, W2 1PG, UK.

64. Center for Life Course Health Research, Faculty of Medicine, University of Oulu, Oulu, FI-90014, Finland.

65. Unit of Primary Care, Oulu University Hospital, Oulu, 90220, Finland.

66. Department of Genomics of Common Disease, School of Public Health, Imperial College London, London, SW7 2AZ, UK.

67. Groningen Research Institute for Asthma and COPD (GRIAC), University of Groningen, University Medical Center Groningen, Groningen, 9700 RB, the Netherlands.

68. Pediatric Research Center, Department of Women´s & Child Health, University of Leipzig, Leipzig, 04103, Germany.

69. IFB Adiposity Diseases, University of Leipzig, Leipzig, 04103, Germany.

70. Department of Child Health, School of Medicine, Cardiff Univeristy, Cardiff, CF14 4XN, UK.

71. Institute of Social and Preventive Medicine, Lausanne University Hospital (CHUV), Lausanne, 1010, Switzerland.

72. Department of Clinical Physiology and Nuclear Medicine, Kuopio University Hospital, Kuopio, FI-70029, Finland.

73. Kuopio Research Institute of Exercise Medicine, Kuopio, FI-70100, Finland.

74. University of North Carolina (UNC) School of Medicine, Chapel Hill, NC 27599, USA.

75. Department of Clinical Chemistry, Fimlab Laboratories, Tampere, 33520, Finland.

76. Department of Clinical Chemistry, University of Tampere School of Medicine, Tampere, 33014, Finland.

77. Li Ka Shing Centre for Health Information and Discovery, The Big Data Institute, University of Oxford, Oxford, OX3 7BN, UK.

78. Research Center for Prevention and Health Capital Region, Center for Sundhed, Rigshospitalet – Glostrup, Copenhagen University, Glostrup, DK-2600, Denmark.

79. Department of Medicine and Therapeutics, The Chinese University of Hong Kong, Hong Kong, Hong Kong, -, China.

80. Li Ka Shing Institute of Health Sciences, The Chinese University of Hong Kong, Hong Kong, Hong Kong, -, China.

81. Hong Kong Institute of Diabetes and Obesity, The Chinese University of Hong Kong, Hong Kong, -, China.

82. Institute of Social and Preventive Medicine, University Hospital of Lausanne, Lausanne, 1010, Switzerland.

83. Estonian Genome Center, University of Tartu, Tartu, 50090, Estonia.

84. Oxford National Institute for Health Research (NIHR) Biomedical Research Centre, Churchill Hospital, Oxford, OX3 7LE, UK.

85. Centre for Genetic Origins of Health and Disease (GOHaD), The University of Western Australia, Crawley, WA 6009, Australia.

86. Department of Clinical Medicine, Copenhagen University, Copenhagen, DK-2200, Denmark.

87. Department of Medicine, Stanford School of Medicine, Stanford, California, CA 94305, USA.

88. Department of Genetics, University of North Carolina, Chapel Hill, NC 27599, USA.

89. Department of Clinical Epidemiology, Leiden University Medical Center, Leiden, 2333 ZA, the Netherlands.

90. Department of Public Health and Primary Care, Leiden University Medical Center, Leiden, 2333 ZA, the Netherlands.

91. Epidemiology Section, BESC Department, King Faisal Specialist Hospital and Research Centre, Riyadh, 12713, Saudi Arabia.

92. Department of Biostatistics, University of Liverpool, Liverpool, L69 3GA, UK.

93. Department of Pediatrics, University of Iowa, Iowa City, IA 52242, USA.

94. Research Unit for Gynaecology and Obstetrics, Department of Clinical Research, University of Southern Denmark, Odense, 5230, Denmark.

95. William Harvey Research Institute, Barts and the London School of Medicine and Dentistry, Queen Mary University of London, London, EC1M 6BQ., UK.

96. Institute of Psychiatry, Kings College London, London, SE5 8AF, UK.

97. Medical Research Council (MRC), Social, Genetic and Developmental Psychiatry Centre, London, SE5 8AF, UK.

98. Department of Paediatrics, University of Cambridge, Cambridge, CB2 0QQ, UK.

99. Wellcome Trust Sanger Institute, Hinxton, Cambridgeshire, CB10 1HH, UK.

100. Department of Tropical Hygiene, Faculty of Tropical Medicine, Mahidol University, Bangkok, 10400, Thailand.

101. Research Centre of Applied and Preventive Cardiovascular Medicine, University of Turku, Turku, 20014, Finland.

102. Max Planck Institute for Psycholinguistics, Nijmegen, 6525 XD, the Netherlands.

103. Department of Clinical Physiology and Nuclear Medicine, Turku University Hospital, Turku, 20520, Finland.

104. BHF Centre for Cardiovascular Science, University of Edinburgh, Queen's Medical Research Institute, Edinburgh, Scotland, EH16 4TJ, UK.

105. Department of Psychology, Mid Sweden University, Östersund, 83125, Sweden.

106. Division of Endocrinology, Department of Medicine, Boston Children's Hospital, Boston, MA 02115, USA.

107. Department of Genetics, Harvard Medical School, Boston, MA 02115, USA.

108. Center for Basic and Translational Obesity Research, Boston Children's Hospital, Boston, MA 02115, USA.

109. Program in Medical and Population Genetics, Broad Institute of Harvard and MIT, Cambridge, MA 02142, USA.

110. Saw Swee Hock School of Public Health, National University of Singapore, National University Health System, Singapore, 119077, Singapore.

111. Singapore Eye Research Institute, Singapore, 168751, Singapore.

112. Department of Obstetrics and Gynecology, Sahlgrenska University Hospital, Gothenburg, N-0403, Sweden.

113. Novo Nordisk Foundation Center for Basic Metabolic Research and Department of Public Health, Faculty of Health and Medical Sciences, University of Copenhagen, Copenhagen, DK-2200, Denmark.

114. Institute of Preventive Medicine, Bispebjerg and Frederiksberg Hospital, The Capital Region, Copenhagen, DK-2000, Denmark.

115. Department of Obstetrics and Gynaecology, University of Cambridge, National Institute for Health Research Cambridge Comprehensive Biomedical Research Centre, Cambridge, CB2 0SW, UK.

116. Population Health Research Institute, St George's University of London, London, Cranmer Terrace, SW17 0RE, UK.

117. IMIM (Hospital del Mar Medical Research Institute), Barcelona, 08003, Spain.

118. Department of Statistics and Applied Probability, National University of Singapore, Singapore, 117546, Singapore.

119. Life Sciences Institute, National University of Singapore, Singapore, 117456, Singapore.

120. Division of Metabolic and Nutritional Medicine, Dr. von Hauner Children's Hospital, University of Munich Medical Center, Munich, 80337, Germany.

121. European Centre for Environment and Human Health, University of Exeter, Truro, TR1 3HD, UK.

122. Department of Internal Medicine, Erasmus MC, University Medical Center Rotterdam, 3015 CE, the Netherlands.

123. Department of Public Health, Academic Medical Center (AMC), University of Amsterdam, Amsterdam, 1100 DD, the Netherlands.

124. Usher Institute for Population Health Sciences and Informatics, University of Edinburgh, Edinburgh, Scotland, EH8 9AG, UK.

125. MRC Human Genetics Unit, Institute of Genetics and Molecular Medicine, University of Edinburgh, Edinburgh, Scotland, EH4 2XU, UK.
